# Individual and household factors associated with tungiasis in a marginalized population in Karamoja, northeastern Uganda

**DOI:** 10.1186/s41182-025-00841-2

**Published:** 2026-03-03

**Authors:** Lynne Elson, Abneel K. Matharu, Berrick Otieno, Hermann Feldmeier, Charles Waiswa, Amina Abubakar, Jürgen Krücken, Ulrike Fillinger, Francis Mutebi

**Affiliations:** 1 KEMRI-Wellcome Trust Research Programme, Kilifi, Kenya; 2 Centre for Tropical Medicine and Global Health, Nuffield Department of Medicine, University of Oxford, Oxford, UK; 3 Institute for Parasitology and Tropical Veterinary Medicine, Freie Universität Berlin, Berlin, Germany; 4 Human Health Theme, International Centre of Insect Physiology and Ecology (ICIPE), Nairobi, Kenya; 5 Institute for Human Development, Aga Khan University, Nairobi, Kenya; 6 Institute of Microbiology, Infectious Diseases and Immunology, Charité University Medicine, Berlin, Germany; 7 College of Veterinary Medicine, Animal Resources and Biosecurity, Makerere University, Kampala, Uganda; 8 Veterinary Centre for Resistance Research, Freie Universität Berlin, Berlin, Germany

**Keywords:** Tungiasis, Neglected tropical diseases, Risk factors, WASH, Disability, Karamoja

## Abstract

**Background:**

Tungiasis is a neglected tropical skin disease caused by adult female fleas which burrow into the skin of people and animals, causing considerable pain and itching. The distribution of the disease is heterogeneous, with Napak district, Karamoja sub-region of northeastern Uganda having the highest disease burden recorded globally. We aimed to determine the factors associated with this high prevalence to inform future intervention strategies.

**Methods:**

We conducted a cross-sectional study to identify factors associated with infection of individual children and adults, and a nested case–control study to identify factors for whole households (families). Infected children were identified through mass-screening of children aged 8–14 years between January and March 2022 in 25 villages. Of the 1619 children screened, 210 infected and 358 uninfected children were randomly selected, and their households were enrolled into the study. Observations were made of the homesteads, and structured interviews were conducted with the caregivers. All adults and children in the households were examined. Mixed effect logistic regression analysis was used to identify factors associated with infection of individuals or households.

**Results:**

We found children who lived in high-density settlements (manyattas) had more than three times the odds of being infected than those in more open, low-density settlements (adjusted odds ratio aOR 3.51, 95% CI 1.57–7.83, *p* = 0.002). To our knowledge, this is the first study to show an association of household infection (at least one case) with having a child with a disability (aOR 5.38, 95% CI 1.92–15.03, *p* = 0.001) and a caregiver who did not show affection to their child (aOR 1.79, 95% CI 1.02–3.13, *p* = 0.041). For individual adults, those who reported drinking alcohol had four times the odds of infection than those who did not (aOR 4.74, 95% CI 1.93–11.68, *p* = 0.001). Frequency of washing feet, soap use and house cleanliness were also associated with household infection.

**Conclusion:**

Control programs should be developed together with the caregivers to enable them to reduce alcohol use, improve their childcare, hygiene and sanitation practices.

**Supplementary Information:**

The online version contains supplementary material available at 10.1186/s41182-025-00841-2.

## Background

Tungiasis has recently received renewed attention due to its addition to the World Health Organization’s list of Neglected Tropical Diseases [1], as a Neglected Tropical Skin Disease targeted for control [2] and now the adoption of a resolution by the 78th World Health Assembly of “Skin Diseases as a Global Public Health Priority” [3]. In the past few years epidemiological [4–7], entomological [8], genomic [9] and intervention studies [10] have been conducted to better understand the ecology and impact [11–13] of the parasite and how it might be controlled.

Tungiasis, also known as sand flea disease or “jiggers” in Africa, is caused by female adult *Tunga penetrans* [14]. The disease is widespread in South America, the Caribbean, and Sub-Saharan Africa, mostly affecting extremely resource poor, marginalized families in rural and peri-urban communities [14]. The adult female flea (imago) burrows into the skin, usually of the feet, and grows 2000-fold in size as the eggs develop in its abdomen [15]. Eggs are expelled into the environment, where larvae develop into pupae and emerge as adults within three weeks under suitable conditions [16]. Off-host development predominantly occurs on earthen floors of sleeping areas [8, 17]. The growing female flea induces a strong inflammatory response which causes considerable pain and itching which in turn cause difficulty in walking, sleeping and concentration in class [11, 13]. Children with tungiasis have been shown to have poor cognitive function [12], lower academic achievement [11], and to experience stigma and discrimination [18–20].

Although it is widespread, the distribution of tungiasis is extremely heterogeneous. In Kenya, national school-based surveys in 2021 estimated a prevalence of 1.35%, with individual schools ranging from < 1% to 22.5% [4]. Community-based surveys elsewhere have reported rates from 7% in a Kenyan village [7] to 62.8% in north-eastern Uganda [21].

To date, several risk factor studies have been conducted in affected communities in different countries in Africa and in Brazil in South America. After age (under 15 years and over 60) and sex (male), most factors identified are related to poverty including living in low standard housing with earthen floors [22–25], not wearing closed shoes [25–27], presence of domestic animals [22, 28, 29], practicing open defecation [22, 25], and not washing feet twice a day with soap [7, 24, 30].

Poverty is clearly strongly associated with tungiasis in most communities, and high prevalence is especially observed in remote, neglected areas. Napak District, in Uganda has historically shown to have extremely high disease prevalence [21], which might in part have to do with the different way of living, the cultural context and behaviors, and such communities have not been studied in depth. Napak district is unusual in being populated by pastoralists, the Karamojong, whose women and children live in high density but rural villages, while the men and adolescent boys tend to be nomadic, herding livestock far from the home for pasture and to avoid violent stock theft [31]. Historically, the area has been marginalized and neglected, and has the highest poverty rate in Uganda [31, 32]. Since the government of Uganda has prioritized tungiasis for control [33], we aimed to determine factors associated with tungiasis among children aged 8–14 years, adults and whole households in Napak District, which may guide prevention programs.

## Methods

### Study design

This exploratory study combined a cross-sectional design for children (8–14 years) and adults (> 18 years) with a case–control study of their households. The surveys were carried out between January and March 2022, the main dry season for the area.

### Study area and population

**Fig. 1:**
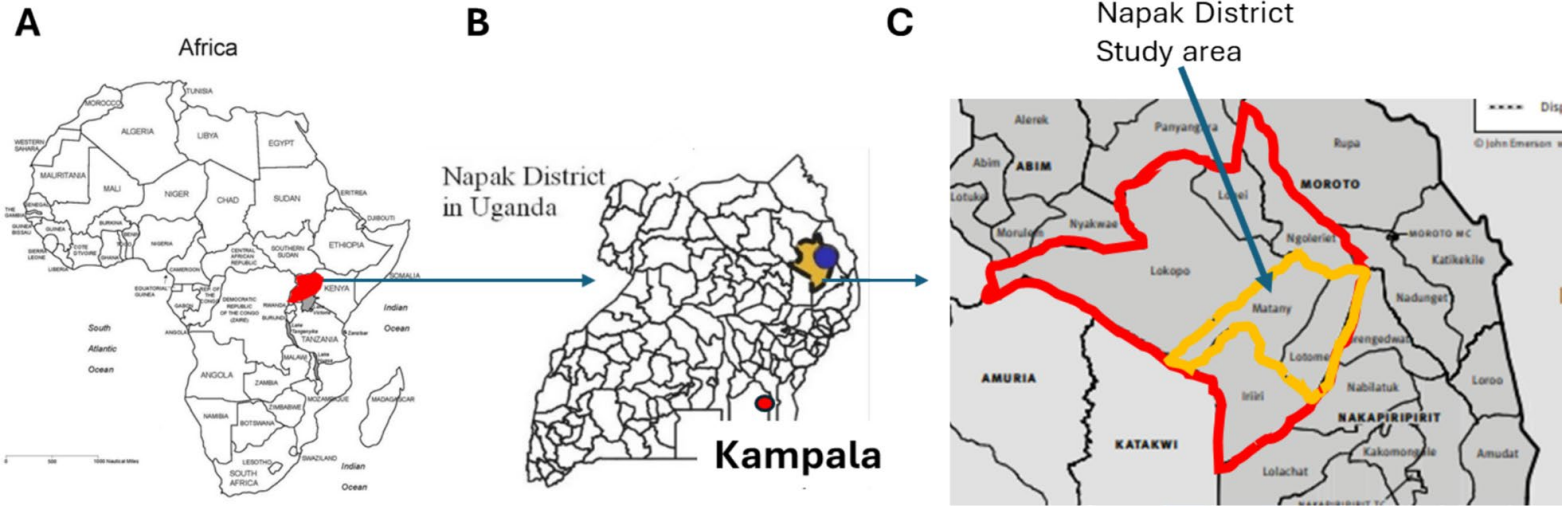
Maps of **A** Africa, **B** Uganda and **C** Napak district in the Karamoja sub-region to show the study sites. **B** Adapted from a map extracted from Mutebi 2023 [21] (cc by 4.0), **C** based on a map extracted from Powell 2010 [31] (permission for use obtained)

**Fig. 2:**
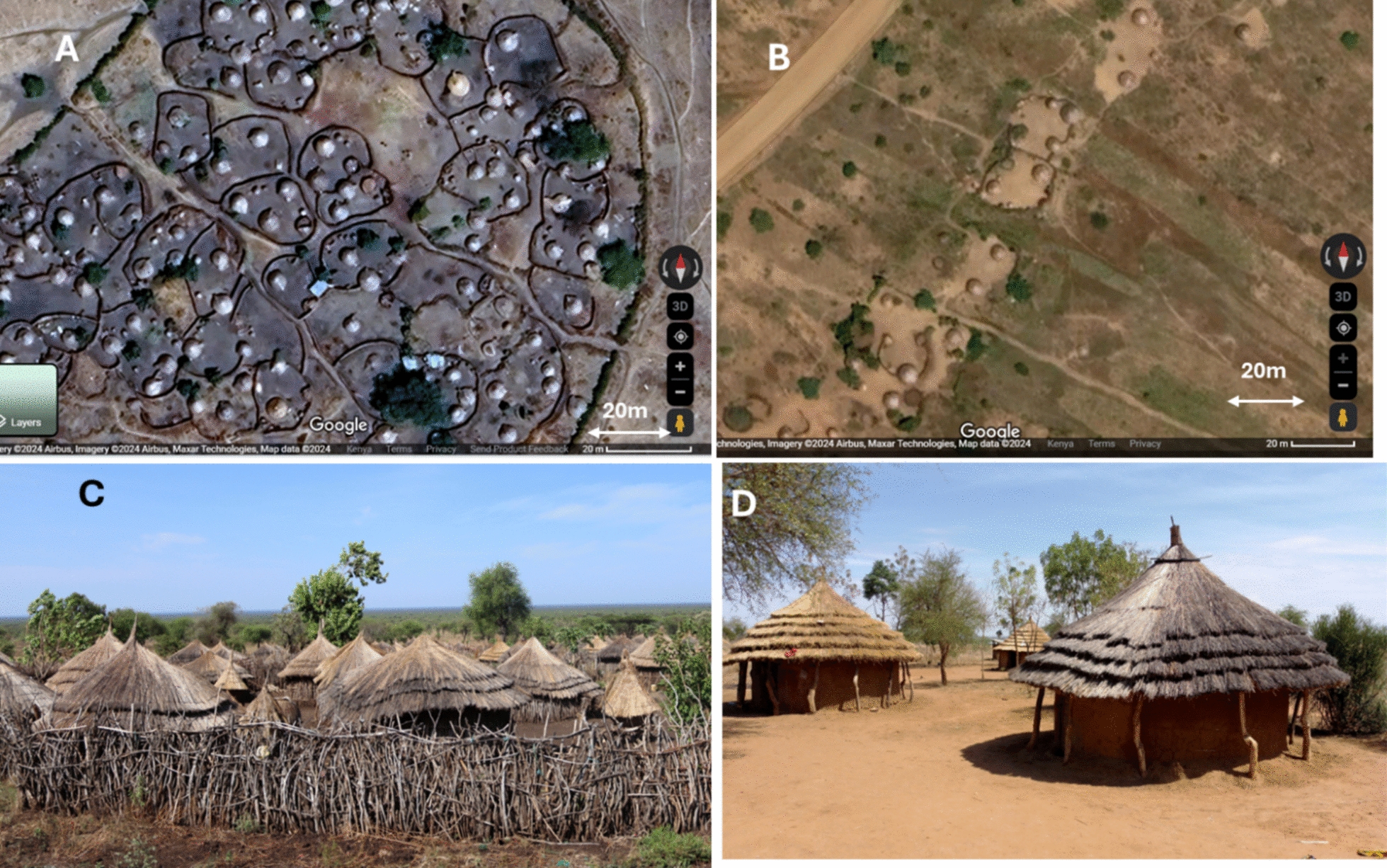
**A** Aerial image of a manyatta taken from Google Earth. **B** Aerial image of homesteads not in a manyatta, taken from Google Earth, **C** a typical manyatta (credit Mutebi), **D** households in an open homestead (credit Mutebi)

The study was conducted in 25 villages in three sub-counties (Lotome, Matany, Lokiteded) of Napak District in northeastern Uganda (Fig. 1). The region is predominantly inhabited by the Karamojong, a pastoralist community practicing transhumance, where men and adolescent boys migrate with livestock in search of pasture and to avoid violent stock theft [31]. Women, children, and the elderly typically reside in permanent, high-density housing clusters enclosed by thorny hedges or stick barriers, known as manyattas. Within each manyatta, crude fencing separates individual homesteads and households (Fig. 2). Households not located in manyattas live in more dispersed homestead clusters (Fig. 2C). For this study, a household was defined as a family unit that shares sleeping structures and meal preparation. Manyattas may contain multiple households, which are not necessarily related.

The Karamojong have long faced threats to their livelihoods, including marginalization, loss of communal grazing land to private development, livestock diseases, desert locust invasions, crop failures, drought, famine, and armed conflict [34–36]. Consequently, this area has the highest poverty rate of the country, the lowest literacy rate among people over 10 years (25% v 74% nationally) [32] and at the time of the study, was experiencing a famine affecting 80% of the population [34].

### Sample size

For the case: control study of households we based our sample size estimate on a previous household risk factor study in Kenya where cases had a twofold higher odds than controls of not washing their feet twice a day and 20% of the controls did not wash their feet twice a day [7]. Thus, we estimated the sample size for an unmatched case–control study assuming a 1:2 ratio of infected (cases) to uninfected (controls) children. With 20% of controls expected to be exposed to not washing their feet twice a day and an odds ratio of 2.0 to be detected at 80% power and 95% confidence, we required 126 cases and 252 controls (OpenEpi Version 3.0). To account for uncertainty in baseline estimates and the inclusion of multiple covariates in multivariable analyses, we inflated the sample size by 30%, yielding a target of approximately 160 case, and 320 control households in Napak district.

### Procedures

Recruitment began in Lotome sub-county, but due to a shortage of control households (those without infected members), additional households were enrolled from villages in Matany and Lokiteded sub-counties, located approximately 80 km from Lotome. Although the populations in these areas belonged to the same ethnic group, they lived in lower-density housing structures (Fig. 2B and D).

In each village, Village Health Teams (VHTs) invited all children aged 8–14 years to a central location for examination. Under supervision, each child washed their feet before trained field officers inspected their hands and feet. Basic demographic data were collected, including age, sex, housing type (manyatta or not), and floor material of the sleeping area (Additional File 1).

Following examination, only children living in houses with earthen floors—a known risk factor for tungiasis [5, 22, 23]—were eligible for selection. These children were stratified into infected (“cases”) and uninfected (“controls”). Where possible, 10 cases and 20 controls were randomly selected per village using a paper lottery. If fewer eligible children were available, all were included.

Households of selected children were then visited for enrollment. Before enrolling control households, the absence of tungiasis among all members was confirmed. Any household found to have an infected member received treatment and was excluded. In enrolled households, all available family members were examined for tungiasis as described in the following section.

### Clinical assessment procedures

Children aged 8–14 years had their height and weight measured. Tungiasis examinations were conducted systematically by inspecting the hands and nine zones on each foot—from the largest to smallest toe, medial and lateral sides, sole, and heel—as previously described [5].

Examination tools are detailed in Additional File 1. For children with embedded sand fleas, infection intensity was assessed by counting: (i) live fleas (round white lesions with a central dark spot or hyperemic painful zones); (ii) dead fleas (black, irregular lesions); (iii) manipulated lesions (where fleas had been removed); and (iv) clusters too dense to count individually, estimated as five fleas per cluster. All participants were also screened for other skin conditions, including fungal infections, scabies, podoconiosis, cutaneous larva migrans, myiasis, and warts.

### Caregiver interviews

Caregiver interviews were conducted at participants’ homes in private settings, followed by guided observations of homestead structures. Demographic data were collected for each participant, including age, sex, relationship to the household head, presence of disabilities or other illnesses, and the building in which they slept. Adults were additionally asked about their education level, marital status, alcohol use, religion, and occupation. Interviews and observations were conducted using structured questionnaires (see Additional File 1), translated into Karamojong and pre-tested in neighboring villages. Trained field officers from the local community carried out all data collection.

### Explanatory variables

We sought to examine both the daily lives of children and the broader circumstances of their households. Given that fleas spend much of their life cycle in the environment [8, 16] and transmission occurs through contact with adult fleas (imagoes), we assessed factors related to housing structure, sanitation, and sleeping arrangements. This included the condition and cleanliness of buildings, and who slept in each room.

Building on prior studies highlighting the role of hygiene [7], we evaluated access to water, soap, and sanitation, as well as foot-washing practices of both the caregiver and the index child. We also considered whether caregivers assisted younger children with hygiene. Because animals can harbor fleas [37] and contribute to environmental contamination, we recorded domestic animal ownership and their housing arrangements. Adult education level was included due to its known influence on child health outcomes [38].

Based on field observations, we also examined whether the index child or other household members had physical or mental disabilities (unrelated to tungiasis), chronic illnesses, or other skin conditions that might increase vulnerability.

Recognizing tungiasis as a disease of neglect, we explored whether this extended to parenting styles—an area not previously studied. We included indicators conceptually linked to neglect, such as whether caregivers knew their child’s friends and their families, how much time they spent with the child, and whether they expressed physical affection, including changes over time.

Children not living with biological parents may be at greater risk of neglect and poorer health outcomes, including tungiasis [39]. Therefore, we explored family structure, the child’s relationship to the household head and caregiver, their preferred confidant when unwell, and whether they felt they received equal food compared to other family members.

### Data analysis

Study data were collected and managed using electronic data capture tools using the REDCap application [40] on handheld devices and data uploaded daily to a password protected server. All analyses were conducted in Stata/BE 18.

For all examined children and adults, the number and proportion (%) infected were calculated. Infection intensity for each individual was calculated as the total number of fleas—live, dead, or manipulated—on both feet, plus five fleas per cluster (used as an estimated average). Cases were classified as mild (≤ 10 fleas) or severe (> 10 fleas), following established criteria [5]. Since infection intensity was not normally distributed, medians and interquartile ranges (IQR) were reported. Differences across age groups were assessed using the Kruskal–Wallis test, and sex differences using the Wilcoxon rank-sum test.

Height-for-age and weight-for-age z-scores were calculated using the “zanthro” extension in Stata, based on UK-WHO Term Growth Charts for adolescents [41]. Children were classified as moderately or severely wasted or stunted using standard z-score thresholds of − 2.0 and − 3.0, respectively.

To assess socioeconomic status (SES), we included household ownership of assets (e.g., phone, radio, bicycle, motorbike, solar power system, land) and income source (occupation). A composite SES variable was generated using polychoric principal component analysis, incorporating ownership of radio, mobile phone, bicycle, motorcycle, solar power, and livestock (see Additional File 2, Section S1). Associations between SES and other explanatory variables were tested using bivariable linear mixed-effects models with village ID as a random effect (results in Additional File 2, Table S2).

Differences in infection status across categorical variables were tested using Chi-squared tests, with *p*-values reported in the population characteristics tables. To identify risk factors for tungiasis, we used three sets of bivariable and multivariable multilevel mixed-effects logistic regression models (see Table 1). Each model included the village unique identifier as a random effect to account for clustering. Variables with *p* < 0.2 in bivariable analysis were included in the full multivariable model. Final models were selected using backward elimination and Akaike Information Criteria (AIC), with Wald tests used to confirm variable significance. Univariable results are presented as odds ratios (OR), and multivariable results as adjusted odds ratios (aOR), with 95% confidence intervals (CI) and *p*-values. Where necessary, categorical variables were collapsed to ensure sufficient sample sizes for analysis.

**Table 1:** Details of the three mixed-effect logistic models run to assess factors associated with tungiasis

Model number	Participants	Data collection tool	Dependent variable	*N*^a^
1	Children aged 8–14 years examined in the villages	Demographics collected during examination	Child had at least one flea vs. no infection	1619
2	Households of selected case and control index children aged 8–14 years	Caregiver questionnaire	Household had at least one case vs. no cases	568
3	All adults examined at the households of selected case and control index children	Demographics collected during examination	Adults had at least one flea vs. no infection	693

^a^ Number of observations included in the final multivariable model

## Results

### Study population characteristics

**Fig. 3:**
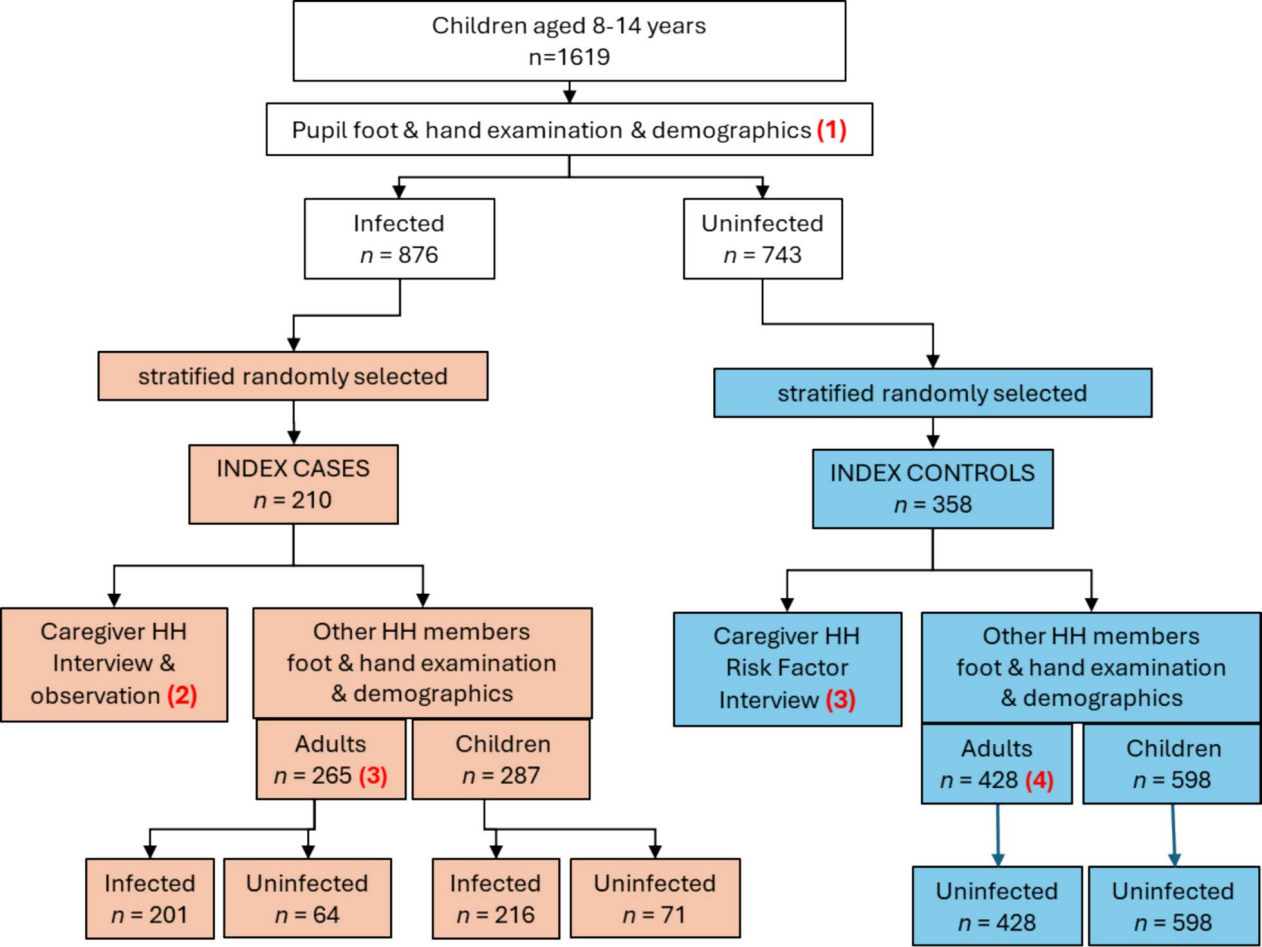
Study flowchart. HH, household

A total of 1619 children aged 8–14 years from 25 villages were examined and just over a half (54.1%, *n* = 876) were found to have tungiasis (Fig. 3) of whom 210 were selected as index cases. Of the uninfected children, 358 were selected as index controls for the household observations and caregiver interviews. Although we aimed for a ratio of 2 controls for every case, due to the very high prevalence we only managed a ratio of 1.7 controls for each case. In the households of index cases, a total of 265 adults and 287 children were examined, of whom 201 (75.8%) adults and 216 (75.3%) children were found to be infected (Fig. 3).

### Infection intensity of all identified cases

Among all the 1298 cases, the median infection intensity was 8 fleas (IQR 3–21). There was no difference in infection intensity between male and female cases, except in the 8–14 years age group where boys had a higher median infection intensity than girls (9 v. 6, Wilcoxon test *p* = 0.004, Additional File Table S2). There was a significant difference in infection intensity between the age groups, (Kruskal–Wallis test *p* = 0.008) with adults over 60 years having the highest (median 18 fleas (IQR 5–38) and the 15- to 24-years age group had the lowest (median 4, IQR 3–6) (Additional File 2 Table S3).

### Demographic factors associated with tungiasis among children aged 8 to 14 years (model 1)

Of the 1619 children examined, 45.7% (739) were boys, 76% (1232) lived in a manyatta, 27.5% (445) were either moderately or severely wasted and 14.4% (233) were moderately or severely stunted (Table 2). Almost a quarter (22.7%, *n* = 367) of all the children examined had another skin condition other than tungiasis, half (51.5%, *n* = 189) of which were identified as “ringworm” (Tinea capitis or Tinea corporis).

**Table 2:** Characteristics of children aged 8–14, by infection status

Variable	Category	All (*n*^a^)	Uninfected (*n*)	Infected *n* (%^b^)	Chi^2^ *p*-value
All		1619	743	876 (54.1)	< 0.001
Sex	Female	880	450	430 (48.9)	
	Male	739	293	446 (60.4)	
Live in a manyatta	No	386	323	63 (16.3)	< 0.001
	Yes	1232	420	812 (65.9)	
Had another skin condition	No	1249	589	660 (52.8)	0.079
	Yes	367	154	213 (58.0)	
Had Ringworm	No	178	69	110 (61.5)	0.196
	Yes	189	86	103 (54.5)	
Floor of room sleep in	Mud/soil	1607	737	870 (54.1)	0.774
	Concrete	12	6	6 (50.0)	
Wasted	No	1174	556	618 (52.6)	0.003
	Moderate	315	146	169 (53.7)	
	Severe	130	41	89 (68.5)	
Stunted	No	1136	643	743 (53.6)	0.035
	Moderate	175	83	92 (52.6)	
	Severe	58	17	41 (70.7)	

^a^ Number, ^b^ percent infected

Children who lived in a manyatta had more than three times the odds of being infected (aOR3.51, 95% CI 1.57–7.83, *p* = 0.002) (Table 3). The odds of boys being infected was nearly twice as high than for girls (aOR 1.85, 95% CI 1.45–2.36, *p* < 0.001), and increasing age was inversely associated with infection. Children who had another skin condition had higher odds of also having tungiasis (aOR 1.43, 95% CI 1.06–1.93, *p* = 0.018). Children who were severely wasted (more than 3.0 standard deviations below the mean) had twice the odds of being infected (aOR 2.12, 95% CI 1.33–3.38, *p* = 0.001).

**Table 3:** Risk factors for tungiasis among children aged 8–14 years (model 1)

			Bivariable				Final multivariable (*n* = 1614)			
Variables	Categories	*N*^a^	OR^b^	95% CI^c^		*P*^d^	aOR	95% CI		*P*
Age		1618	0.88	0.83	0.94	< 0.001	0.89	0.83	0.95	< 0.001
Sex	Female	880	1				1			
	Male	739	1.89	1.49	2.4	< 0.001	1.85	1.45	2.36	< 0.001
Had another skin infection	No	1249	1				1			
	Yes	367	1.53	1.14	2.06	0.004	1.43	1.06	1.93	0.018
Wasted	No	1174	1				1			
	Moderate	315	1.19	0.89	1.6	0.245	1.02	0.75	1.39	0.888
	Severe	130	2.46	1.56	3.88	< 0.001	2.12	1.33	3.38	0.001
Stunted	No	1136	1							
	Moderate	175	1.15	0.78	1.67	0.480				
	Severe	58	1.67	0.83	3.37	0.153				
Live in a manyatta	No	386	1				1			0.002
	Yes	1232	4.26	1.95	9.3	< 0.001	3.51	1.57	7.83	

^a^ Number, ^b^ odds ratio, ^c^ confidence interval, ^d^ *p*-value, ^e^ adjusted odds ratio

### Household risk factors (model 2)

Due to men spending most of their time herding livestock away from home, the majority of respondents were female, with 90.7% (*n* = 515) identifying as both head of household and primary caregiver (Table 4). Although 73% of households had access to water via a shared borehole, sanitation remained poor: 80.3% practiced open defecation and 66.4% disposed of waste indiscriminately. Most households (93.4%) had only one sleeping room in the main house, shared by adults and children and 74.7% (*n* = 424) of index children slept in this room. Approximately one-third (31.5%) of households lived in shared compounds with other families, and 64.8% were located within a manyatta. The distribution of all explanatory variables by household infection status (case or control) is included in Additional file 2 Table S4.

**Table 4:** Household characteristics by infection status

Variable	Category	All (*n*)	Controls (*n*)	Cases (*n*, %)	Chi^2^ *p*-value
All		568	358	210 (37.1)	
Median HH size (IQR)		5 (4–6)	5 (4–6)	5 (4–6)	
HH^1^ in a manyatta	No	200	190	10 (5.0)	< 0.001
	Yes	368	168	200 (54.4)	
HH in a shared homestead	No	389	256	133 (34.2)	0.042
	Yes	179	106	77 (43.0)	
Sex index child	Male	230	127	103 (44.8)	0.002
	Female	339	231	108 (31.9)	
Sex HH head	Male	54	41	13 (24.1)	0.038
	Female	515	317	198 (38.5)	
Sex caregiver	Male	32	19	13 (40.6)	0.669
	Female	537	339	198 (36.9)	
Adult over 60 years in HH	No	502	325	177 (35.3)	0.020
	Yes	66	33	33 (50.0)	
Land status	Not owned	185	114	71 (38.4)	0.657
	Owned	384	244	140 (36.5)	
Water source	piped or own well	31	30	1 (3.2)	< 0.001
	Shared borehole	415	216	199 (48.0)	
	Unimproved/open	119	110	9 (7.6)	
Toilet	Latrine	111	71	40 (36.0)	0.808
	Open defecation	456	286	170 (37.3)	
Waste disposal	Discarded anywhere	377	236	141 (37.4)	0.471
	In a pit	55	39	16 (29.1)	
	Collected and burned	130	81	49 (37.7)	
Number of meals yesterday	0 or 1	383	219	164 (42.8)	< 0.001
	2 or 3	185	139	47 (24.9)	
Income source	Employed, other	13	11	2 (15.4)	0.002
	Casual labor	367	219	148 (40.3)	
	Farming	32	25	7 (21.9)	
	Selling goods	76	59	17 (22.4)	
	None	74	40	34 (46.0)	
Brew their own alcohol	No	366	225	141 (38.5)	0.302
	Yes	202	133	69 (34.2)	

^1^ Household

In spite of exploring many potential risk factors (Additional file 2, Table S4) only six remained in the final multivariable model (Table 5). Although relatively uncommon (36 households), having an index child with a disability was strongly associated with household infection, with affected households having over five times the odds of infection compared to those without a disabled child (aOR 5.38, 95% CI 1.92–15.03, *p* = 0.001).

Caregivers in most households (*n* = 521) reported that their index child did not consistently use soap when washing their feet. These households had nearly five times the odds of tungiasis infection compared to the few (*n* = 28) where the child always used soap (aOR 4.73, 95% CI 1.11–20.19, *p* = 0.036). Additionally, households where caregivers washed their own feet less than twice daily had twice the odds of infection compared to those reporting twice-daily washing (aOR 2.39, 95% CI 1.29–4.42, *p* = 0.006).

Household cleanliness also played a role. In 63.6% of case households, the index child’s sleeping room—typically the main house—was unswept and cluttered, compared to 37.7% of control households. An untidy sleeping room was associated with twice the odds of infection (aOR 2.09, 95% CI 1.22–3.60, *p* = 0.007).

As a possible indicator of parenting style and neglect, caregivers were asked if they hug their child. Nearly half of them (49%) said they do not hug their child, and these households had twice the odds of being infected than those where the caregiver said they do hug the child (aOR 1.79, 95% CI 1.02–3.13, *p* = 0.041).

In bivariable linear regression analysis for factors associated with SES (Additional file 2 Table S5), we found households situated within a manyatta had a lower SES than those that were not (β − 0.16, 95% CI − 0.24 to − 0.09, *p* < 0.001). Households who brewed alcohol at home had a higher SES than those who did not (β 0.16, 95% CI 0.11–0.20, *p* < 0.001). The only variable that correlated with economic status that was also associated with tungiasis was the brewing of alcohol at home. The 35% of households who brewed alcohol at home had half the odds of infection than those that did not brew alcohol (aOR 0.52, 95% CI 0.29–0.93, *p* = 0.029).

**Table 5:** Household risk factors for tungiasis (model 3).^a^

			Full multivariable (*n* = 543)				Final multivariable (*n* = 559)			
Variables	Categories	*N*^b^	aOR^c^	95% CI^d^		*P*^e^	aOR	95% CI		*P*
Index child sex	Female	339	1							
	Male	230	0.45	0.24	0.84	0.012				
HHH^f^ age			0.99	0.95	1.03	0.648				
Caregiver age			1.01	0.98	1.05	0.417				
Index child relationship to caregiver	Child	432	1							
	Other	136	1.00	0.35	2.92	0.994				
Socioeconomic status			1.25	0.30	5.27	0.761				
Brew alcohol at home	No	366	1				1			
	Yes	202	0.75	0.37	1.53	0.435	0.52	0.29	0.93	0.028
Number of jerricans water used daily	0–1.5	154	1							
	1.5–3	327	0.88	0.39	1.98	0.760				
	> 3	87	1.05	0.38	2.91	0.924				
Caregiver foot wash frequency	Twice a day	344	1				1			
	Less often	224	2.59	1.23	5.44	0.012	2.39	1.29	4.42	0.006
Caregiver soap-use for foot washing	Always	54	1							
	Not always	513	0.69	0.21	2.29	0.540				
Number of meals eaten yesterday	0 or 1	383	1							
	2 or 3	185	0.84	0.43	1.65	0.616				
Toilet used	Latrine	111	1							
	Open defecation	456	1.46	0.67	3.17	0.342				
Index child relationship to HH head	Child	432	1							
	Grandchild	63	1.90	0.41	8.93	0.415				
	Other	73	3.06	0.87	10.79	0.082				
Index child disability	No	532	1				1			
	Yes	36	6.66	2.17	20.50	0.001	5.38	1.92	15.03	0.001
Frequency wash young children	Twice a day	329	1							
	Once a day	121	1.42	0.58	3.51	0.442				
	Less often	64	2.12	0.70	6.42	0.184				
	Never	52	1.61	0.55	4.71	0.385				
Index child frequency wash feet	Twice a day	388	1							
	Less often	159	0.76	0.30	1.93	0.563				
	Don’t know	21	2.83	0.11	71.04	0.527				
Index child soap use for foot washing	Always	28	1				1			
	Less often	521	4.08	0.63	26.40	0.140	4.73	1.11	20.19	0.036
	Don’t know	19	0.39	0.01	16.54	0.619	3.00	0.36	24.71	0.308
Caregiver hugged index child	Yes	287	1				1			
	No	278	1.72	0.92	3.23	0.090	1.79	1.02	3.13	0.041
Caregiver education	None	395	1							
	Some	164	0.93	0.45	1.92	0.851				
Family income source	Selling goods^g^	76	1							
	None	74	1.03	0.26	4.09	0.969				
	Casual labor	367	2.36	0.82	6.81	0.112				
	Farming^h^	32	1.46	0.23	9.10	0.688				
	Employed, other	13	0.52	0.04	6.22	0.698				
State of repair main house	Good	373	1							
	Poor	155	1.42	0.71	2.81	0.319				
	Very poor (holes in walls & roof)	33	2.38	0.58	9.78	0.229				
Index child room sanitation	Swept & tidy	277	1							
	Not swept, items scattered	266	1.79	0.90	3.56	0.097	2.09	1.22	3.60	0.007
	Other	19	0.37	0.05	2.51	0.309	0.42	0.08	2.09	0.289
Floor in index child sleep room	Hard clay	398	1							
	Loose clay/soil	155	1.33	0.61	2.94	0.474				
	Other	15	0.23	0.02	2.41	0.219				

^a^ Univariable model in Additional file Table S6; ^b^ number of households; ^c^ adjusted odds ratio; ^d^ confidence interval; ^e^ *p*-value; ^f^ head of household; ^g^ includes selling food, alcohol, etc.; ^h^ crops or livestock

### Risk factors for adults examined in index households (model 3)

The distribution of adults by their own infection status and each variable is detailed in Table 6. Of all the adults examined, 71% reported they were the head of their household, 84% were female and half were between 25 and 44 years old. The level of education of adults was very low, with more than two thirds (68.4%) having had no education.

**Table 6:** Demographic characteristics of adults examined at the households

Variable	Category	All (%)	Uninfected	Infected (%)	*p*-value ^a^
All		693	488	205 (29.6)	
Live in a manyatta	No	248 (35.8)	238	10 (4.0)	< 0.001
	Yes	440 (63.5)	247	193 (43.9)	
Sex	Female	583 (84.1)	407	176 (30.2)	0.420
	Male	110 (15.9)	81	29 (26.4)	
Age groups	18–24	95 (13.7)	83	12 (12.6)	< 0.001
	25–44	352 (50.8)	268	84 (23.9)	
	45–60	160 (23.1)	91	69 (43.1)	
	> 60	86 (12.4)	46	40 (46.5)	
Education level	None	474 (68.4)	309	165 (34.8)	< 0.001
	Some primary	151 (21.8)	124	27 (17.9)	
	More	68 (9.8)	55	13 (19.1)	
Relationship to HH head	Self	489 (71.0)	346	143 (29.2)	0.088
	Spouse	81 (11.7)	59	22 (27.1)	
	Child	48 (6.9)	38	10 (20.8)	
	Sibling	18	12	6 (33.3)	
	Parent	26	12	14 (53.9)	
	Other	31	21	10 (32.3)	
Marital status	Married	540 (77.9)	396	144 (26.7)	0.002
	Other	153 (22.1)	92	61 (39.9)	
Occupation	None	116 (16.7)	67	49 (42.2)	< 0.001
	Casual labor	423 (61.0)	289	134 (31.7)	
	Farming	46 (6.6)	37	9 (19.6)	
	Selling goods	90 (13.0)	79	11 (12.2)	
	Other	18 (2.6)	16	2 (11.1)	
Disability	No	633 (91.3)	460	173 (27.3)	< 0.001
	Yes	60 (8.7)	28	32 (53.3)	
Had another illness	No	397 (57.3)	303	111 (37.5)	< 0.001
	Yes	296 (42.7)	185	94 (23.7)	
Alcohol use	No	106 (15.3)	82	24 (22.6)	0.089
	Yes	587 (84.7)	406	181 (30.8)	
Building usually sleep in	Main house	621 (89.6)	440	181 (29.2)	0.032
	Kitchen	2 (0.3)	0	2	
	Separate hut	31 (4.5)	25	6 (19.4)	
	Other	39 (5.6)	23	16 (43.9)	

^a^ p-value from Chi^2^ test

The adult model was complicated by strong correlations between socioeconomic status (SES) and several variables, including age, education, marital status, occupation, and residence in a manyatta (Additional File 2, Table S5). Consequently, SES and manyatta were excluded from the final model to reduce collinearity, while the remaining variables were retained.

Adults who reported having no occupation had significantly higher odds of tungiasis infection (aOR 5.86, 95% CI 1.86–18.50, *p* = 0.003), as did those engaged in casual labor (aOR 4.89, 95% CI 1.82–12.67, *p* = 0.001), compared to those selling goods (Table 7). Age was also associated with infection: adults aged 45–60 years had nearly five times the odds of infection compared to those aged 18–24 (aOR 4.88, 95% CI 1.93–12.32, *p* = 0.001), and those over 60 had similarly elevated odds (aOR 4.71, 95% CI 1.56–14.18, *p* = 0.006).

Alcohol consumption was common, with 85% of adults reporting use. These individuals had five times the odds of infection compared to those who abstained (aOR 4.74, 95% CI 1.93–11.68, *p* = 0.001). Adults reporting another illness also had increased odds of infection (aOR 2.30, 95% CI 1.35–3.92, *p* = 0.002).

**Table 7:** Risk factors for tungiasis among adults in Napak (model 3)

			Bivariable				Final multivariable (*n* = 693)			
Variable	Categories	*N*^a^	OR^b^	95% CI^c^		*P*^d^	aOR^e^	95% CI		*P*
Sex	Female	583	1							
	Male	110	0.67	0.36	1.23	0.197				
Adult age groups	18–24	95	1				1			
	25–44	352	2.45	1.06	5.68	0.037	2.13	0.88	5.16	0.092
	45–60	160	5.88	2.43	14.23	< 0.001	4.88	1.93	12.32	0.001
	> 60	86	6.81	2.54	18.25	< 0.001	4.71	1.56	14.18	0.006
Level of education	Complete primary or above	68	1							
	None	474	1.94	0.90	4.19	0.090				
	Some primary	151	0.69	0.29	1.67	0.412				
Marital status	Married	540	1							
	Other	153	1.58	0.95	2.63	0.076				
Occupation	Sales of goods	90	1				1			
	None	116	7.98	2.78	22.86	< 0.001	5.86	1.86	18.50	0.003
	Casual labor	423	5.02	1.97	12.83	0.001	4.81	1.82	12.67	0.001
	Farming	46	2.14	0.56	8.25	0.267	1.31	0.31	5.55	0.714
	Other	18	1.28	0.16	9.94	0.815	1.12	0.11	11.19	0.923
Disability	No	633	1							
	Yes	60	1.67	0.81	3.45	0.167				
Alcohol use	No	106	1				1			
	Yes	587	3.96	1.79	8.79	0.001	4.74	1.93	11.68	0.001
Have another illness	No	397	1				1			
	Yes	296	2.42	1.48	3.94	< 0.001	2.30	1.35	3.92	0.002
SES^f^		688	0.20	0.08	0.54	0.001				
Live in a manyatta	No	248	1							
	yes	440	1.92	0.49	7.48	0.346				

^a^ Number, ^b^odds ratio, ^c^ confidence interval, ^d^ *p*-value, ^e^ adjusted odds ratio, ^f^ socioeconomic status

## Discussion

In this study, we set out to determine what factors might be associated with tungiasis in Napak District of the Karamoja sub-region where there was an extremely high prevalence of tungiasis [21], which could be used to inform the design of future intervention programs.

We identified three factors that were associated with tungiasis which to our knowledge have not been reported before; for children this was living in a manyatta and for a household it was having a child with a disability and for adult infection it was drinking alcohol. Factors which have previously been identified and held true in this unique agro-pastoralist community included nutrition status, hygiene practices and sanitation of the child’s room, having another skin disease, age and sex of the child, and SES of their family.

One of the newly identified risk factors for children, which is unique to this part of the world, was living in the manyatta settlement structure. There are two plausible reasons why living in manyattas may be associated with infection in children, one is the lower SES of households within manyattas compared to those not in manyattas which we present in Additional file 2, table S5. Tungiasis has long been considered a disease of poverty and in this study and a previous study [4], it has been demonstrated using an asset score, that tungiasis infection in children is associated with low SES of their household. Another possible cause of the association could be the high population density in manyattas compared to other villages, as illustrated in Fig. 2. High population density likely increases the odds of someone in the village having an infection and higher odds of other people coming into contact with free-living imago female fleas in the soil. In this way, tungiasis is like many infectious and neglected tropical diseases, such as tuberculosis, malaria, dengue, diarrheal diseases, soil transmitted helminths and schistosomiasis, where prevalence or odds of infection is highest among high-density, resource-poor communities[42, 43].

People with disabilities in affected communities tend to have a high infection intensity and extremely severe pathology [44]. In the current study only 36 index children had a disability, and their infection intensity was not different to other cases, but their household had a higher odds of infection than those whose child was not disabled. This was not a formal clinical assessment of disability but reported by the caregivers responding to the household interviews and interviewer observations. People with disabilities (both physical or mental) tend to sit or lie on the ground for extended periods every day, where they would be very vulnerable to host-seeking adult female sand fleas. For the household infection, the higher odds may be due to the infected disabled person increasing the density of off-host stages inside the house as well as increased stress of caring for the disabled person, the extra financial burden or a lower income for the family. It is also possible the disabled child was infected because other family members brought it into the home where the disabled child was very vulnerable to being infected. This factor needs to be investigated further where more people with disabilities are enrolled.

Another risk factor for children aged 8–14 years was their nutritional status. Severe stunting and wasting were both associated with tungiasis, but since they were also associated with each other, only wasting was retained in the final model. This region experiences chronic food shortages and at the time of the survey the whole region was experiencing famine as a culmination of many factors [34, 45]. Our data show 68% of households had only had one meal the previous day. A previous study in Kenya recently also demonstrated an association of tungiasis with malnutrition [11]. It is most likely that tungiasis and child wasting co-occur as a result of poverty and related living conditions and is not a causal relationship. Poverty exposes people both to the conditions which are favorable to transmission of *Tunga penetrans* such as earthen floors and poor hygiene and sanitation, as well as poor access to nutritious food.

Several previous studies have identified poor hygiene practices to be a risk factor for tungiasis [4, 6, 7, 24, 30], both frequency of washing feet and use of soap when doing so. In the current study, households where the caregiver reported that the index child did not always use soap had five times higher odds of infection, and households where the caregiver herself washed her feet less than twice a day also had higher odds of infection. The causal link with hygiene is unknown but it is conceivable that regular washing of feet could remove fecal residue from the embedded fleas and so reduce odors which are attractive to other host-seeking fleas. The physical rubbing action involved in feet-washing may also remove attached fleas before they fully penetrate the skin.

Household tungiasis infection was also found to be associated with the state of cleanliness of the index child’s sleeping room, often the only room of the house shared with other family members, which has been reported to be a risk factor in a few previous studies [22, 25]. Dirty floors with items scattered over them likely provide appropriate conditions, such as organic matter and undisturbed loose soil, for egg hatching, the survival and development of flea larvae and pupae.

Tungiasis in both children and adults was associated with the presence of other skin conditions and illnesses, respectively. These comorbidities likely co-occur within households due to low socioeconomic status, poor hygiene, and inadequate nutrition. Tungiasis may make children more susceptible to other skin infections of the feet due to the open lesions created by the embedded fleas and the associated pathology [15, 46]. For instance, secondary bacterial infections are common in tungiasis [47, 48]. Moreover, tungiasis prevalence was higher in manyattas, where high population density may facilitate transmission of other contact-dependent infections, such as ringworm.

Alcohol use has never been reported as a risk factor for tungiasis before. Here we found it associated with infection of adults, but their consumption was not associated with the infection status of their household. In Karamoja alcohol use is widespread (85% of the adults examined for tungiasis), and is considered to be a cultural norm, with the drinks often being brewed by the household (36% of households in this study), from sorghum and millet. Mostly these are of low alcohol content, and are consumed as a food particularly in times of food insecurity, even by children [35]. Alcohol consumption is usually a social activity and leads people to sit for extended periods in communal areas, with at least their feet on the ground where they are exposed to host-seeking sand fleas. In addition, adults who consume alcohol may neglect their personal hygiene. In contrast, the brewing of alcohol at home was associated with lower odds of infection in a household, likely because they are selling it and had a higher SES, as we show in Additional file 2, table S5.

## Study limitations

There were a few limitations to this study. Firstly, the challenge faced with identifying sufficient numbers of control households who had no infected family members. This forced us to include villages distant from our original study site and with lower density village structures. Secondly, sampling from within clustered villages could have introduced bias in the outcomes. To counter both possible sources of bias we used mixed-effect models using the unique village identifier as a random effect. Another limitation of the household case: control study was that many variables had few observations in some categories, leading to rather wide confidence intervals and therefore limiting our ability to draw strong conclusions for some of the factors that might be promoting the unchecked spread of infections in this area, including child disability, caregiver and child soap-use for washing feet. Lastly, conclusions regarding the adult risk factors mostly apply to women due to the low numbers of men enrolled in the study, and no conclusions can be drawn regarding gender differences.

## Conclusions

The very high prevalence observed in northeastern Uganda is likely the result of multiple compounding factors, including extremely high-density settlements with earthen house floors, widespread poverty, poor hygiene and sanitation practices and limited parental care. Children with disabilities living under these conditions are at particularly high risk of infection. In the short term, it is unlikely that settlement structures can be altered, as they were established to provide protection against the recurrent violence in the area. Therefore, intensive control programs are urgently needed, employing an integrated, community-based approach that combines regular treatment of affected individuals and house floors with health promotion efforts aimed at improving foot-hygiene, encouraging soap-use, reducing alcohol consumption and strengthening childcare practices. Such comprehensive interventions are essential to interrupt the transmission cycle and eliminate the parasites from homesteads.

## Supplementary Information

Supplementary Material 1

Supplementary Material 2.

Supplementary Material 3.

Supplementary Material 4.

Supplementary Material 5.

## Data Availability

The datasets supporting the conclusions of this article are available in the Additional files associated with this manuscript. Additional file 3. Elson Karamoja children 8–14 foot exam dataset.xlsx Description: Microsoft excel workbook containing data and associated codebook for data collected January and March 2022 during examination of children in Karamoja, northeastern Uganda. Contains 62 variables for 1619 observations, 476 Kb Additional file 4. Elson Karamoja HH Risk Factor dataset.xlsx Description: Microsoft excel workbook containing data and associated codebook for data collected January and March 2022 during interviews of caregivers in Karamoja, northeastern Uganda. Contains 162 variables for 568 observations, 488 Kb. Additional file 5. Elson Karamoja adult foot exam dataset.xlsx Description: Microsoft excel workbook containing data and associated codebook for data collected January and March 2022 during examination of adults in Karamoja, northeastern Uganda. Contains 67 variables for 673 observations, 213 Kb.

## Abbreviations

aOR: Adjusted odds ratio
AIC: Akaike information coefficient
CI: Confidence interval
HH: Household
IQR: Inter-quartile range
N: Number
OR: Odds ratio
SES: Socioeconomic status
