# Supplementary material for: Individual and household factors associated with tungiasis in a marginalized population in Karamoja, northeastern Uganda
Source: Trop Med Health. 2026 Mar 3;54:36. doi: 10.1186/s41182-025-00841-2 (PMC12954993; doi:10.1186/s41182-025-00841-2)
Supplement: Supplementary file 1 — Supplementary Material 1 [file 41182_2025_841_MOESM1_ESM.zip › Elson Karamoja tungiasis adult foot exam.pdf]

Adult Household Foot Exam And Flea Count

ID

____

Date

____

Enumerator (interviewer) initials

____

Region

- ◯ BU
- ◯ NA
- ◯ SI
- ◯ KW

Sub County

- ◯ Ngoriet
- ◯ Iriri
- ◯ Lokopo
- ◯ Lotome
- ◯ Matany
- ◯ Lopeei
- ◯ Lorengecora
- ◯ Matany T/C
- ◯ Kangole T/C
- ◯ Lorengecora T/C
- ◯ Others

Other sub county (specify)

____

Parish

____

Village

____

Home Mannyatta

____
(Please indicate "not applicable" if the household is not located within a mannyatta)

School/Village ID number (as per school/village list) from which index child selected

____
(e.g. 200)

Index Child registration number (001-102) as allocated in school/village

____
(number 001 to 102)

Name of Index child (first and family name)

____

Assigned Label for Adult who has feet screened for jiggers

____
(e.g KW_093_001_H_A01)

Name adult (first and family name)	______________________
Age of adult	______________________
Sex of Adult	◯ Male<br>◯ Female
Education level achieved	◯ None,<br>◯ Some primary,<br>◯ Complete primary,<br>◯ Some secondary,<br>◯ Complete secondary,<br>◯ Further
Relationship to household head	◯ Self,<br>◯ Wife,<br>◯ Child,<br>◯ Sibling,<br>◯ Parent,<br>◯ Other-specify
Specify other relation to household head	______________________
Marital status	◯ married monogamous<br>◯ married polygamous<br>◯ widowed<br>◯ single<br>◯ separated/ divorced
Occupation	◯ Full time employee<br>◯ Part time employee<br>◯ Casual labourer<br>◯ Self-Farmer, crop<br>◯ Self-Farmer, livestock<br>◯ Self-Fisherman,<br>◯ Self-selling food or other small-scale business<br>◯ Self-Selling alcohol<br>◯ None<br>◯ Other
Other occupation	______________________
Religion	◯ Muslim,<br>◯ Christian,<br>◯ Traditionist,<br>◯ None,<br>◯ Other (specify)
other religion	______________________

Disability (observe)	◯ Physical,<br>◯ Mental,<br>◯ Both,<br>◯ None.
Current illness	☐ none<br>☐ Respiratory, chest, cough, nose<br>☐ diarrhea, stomach<br>☐ skin rashes,<br>☐ eye problems<br>☐ ear problems<br>☐ headache<br>☐ Fever, malaria<br>☐ Others
Other current illness	______________________
Alcohol use.	◯ Every day,<br>◯ a few times week,<br>◯ once a week,<br>◯ Less,<br>◯ Never
Location of sleeping place	◯ main house,<br>◯ kitchen house,<br>◯ teenage boys house/separate hut,<br>◯ other - specify
other sleeping place	______________________
Tungiasis infection on hands	◯ Yes<br>◯ No
Number of lesions on hands	______________________
Tungiasis infection on feet	◯ Yes<br>◯ No

RIGHT FOOT: TOE 1

Live Fleas	______________________
Dead Fleas	______________________
Manipulated Lesions	______________________
Cluster	______________________
Peeling Skin	◯ Yes ◯ No

Cracks	◯ Yes ◯ No
Ulcers	◯ Yes ◯ No
Abscess	◯ Yes ◯ No
Hard skin	◯ Yes ◯ No
Thick skin around nails	◯ Yes ◯ No
Deformation of nails	◯ Yes ◯ No
Loss of nails	◯ Yes ◯ No

RIGHT FOOT: TOE 2

Live Fleas	__________________________
Dead Fleas	__________________________
Manipulated Lesions	__________________________
Cluster	__________________________
Peeling Skin	◯ Yes ◯ No
Cracks	◯ Yes ◯ No
Ulcers	◯ Yes ◯ No
Abscess	◯ Yes ◯ No
Hard skin	◯ Yes ◯ No
Thick skin around nails	◯ Yes ◯ No
Deformation of nails	◯ Yes ◯ No
Loss of nails	◯ Yes ◯ No

RIGHT FOOT: TOE 3

Live Fleas	______________________
Dead Fleas	______________________
Manipulated Lesions	______________________
Cluster	______________________
Peeling Skin	◯ Yes ◯ No
Cracks	◯ Yes ◯ No
Ulcers	◯ Yes ◯ No
Abscess	◯ Yes ◯ No
Hard skin	◯ Yes ◯ No
Thick skin around nails	◯ Yes ◯ No
Deformation of nails	◯ Yes ◯ No
Loss of nails	◯ Yes ◯ No

RIGHT FOOT: TOE 4

Live Fleas	______________________
Dead Fleas	______________________
Manipulated Lesions	______________________
Cluster	______________________
Peeling Skin	◯ Yes ◯ No
Cracks	◯ Yes ◯ No
Ulcers	◯ Yes ◯ No
Abscess	◯ Yes ◯ No
Hard skin	◯ Yes ◯ No

Thick skin around nails	◯ Yes ◯ No
Deformation of nails	◯ Yes ◯ No
Loss of nails	◯ Yes ◯ No

RIGHT FOOT: TOE 5

Live Fleas	________________
Dead Fleas	________________
Manipulated Lesions	________________
Cluster	________________
Peeling Skin	◯ Yes ◯ No
Cracks	◯ Yes ◯ No
Ulcers	◯ Yes ◯ No
Abscess	◯ Yes ◯ No
Hard skin	◯ Yes ◯ No
Thick skin around nails	◯ Yes ◯ No
Deformation of nails	◯ Yes ◯ No
Loss of nails	◯ Yes ◯ No

RIGHT FOOT: MEDIAL SIDE

Live Fleas	________________
Dead Fleas	________________
Manipulated Lesions	________________
Cluster	________________
Peeling Skin	◯ Yes ◯ No

Cracks	◯ Yes ◯ No
Ulcers	◯ Yes ◯ No
Abscess	◯ Yes ◯ No
Hard skin	◯ Yes ◯ No

RIGHT FOOT: LATERAL SIDE

Live Fleas	________________
Dead Fleas	________________
Manipulated Lesions	________________
Cluster	________________
Peeling Skin	◯ Yes ◯ No
Cracks	◯ Yes ◯ No
Ulcers	◯ Yes ◯ No
Abscess	◯ Yes ◯ No
Hard skin	◯ Yes ◯ No

RIGHT FOOT: HEEL

Live Fleas	________________
Dead Fleas	________________
Manipulated Lesions	________________
Cluster	________________
Peeling Skin	◯ Yes ◯ No
Cracks	◯ Yes ◯ No
Ulcers	◯ Yes ◯ No

Abscess	◯ Yes ◯ No
Hard skin	◯ Yes ◯ No

RIGHT FOOT: SOLE

Live Fleas	__________________
Dead Fleas	__________________
Manipulated Lesions	__________________
Cluster	__________________
Peeling Skin	◯ Yes ◯ No
Cracks	◯ Yes ◯ No
Ulcers	◯ Yes ◯ No
Abscess	◯ Yes ◯ No
Hard skin	◯ Yes ◯ No

LEFT FOOT: TOE 1

Live Fleas	__________________
Dead Fleas	__________________
Manipulated Lesions	__________________
Cluster	__________________
Peeling Skin	◯ Yes ◯ No
Cracks	◯ Yes ◯ No
Ulcers	◯ Yes ◯ No
Abscess	◯ Yes ◯ No
Hard skin	◯ Yes ◯ No

Thick skin around nails	◯ Yes ◯ No
Deformation of nails	◯ Yes ◯ No
Loss of nails	◯ Yes ◯ No

LEFT FOOT: TOE 2

Live Fleas	______________________
Dead Fleas	______________________
Manipulated Lesions	______________________
Cluster	______________________
Peeling Skin	◯ Yes ◯ No
Cracks	◯ Yes ◯ No
Ulcers	◯ Yes ◯ No
Abscess	◯ Yes ◯ No
Hard skin	◯ Yes ◯ No
Deformation of nails	◯ Yes ◯ No
Thick skin around nails	◯ Yes ◯ No
Loss of nails	◯ Yes ◯ No

LEFT FOOT: TOE 3

Live Fleas	______________________
Dead Fleas	______________________
Manipulated Lesions	______________________
Cluster	______________________
Ulcers	◯ Yes ◯ No

Peeling Skin	◯ Yes ◯ No
Cracks	◯ Yes ◯ No
Abscess	◯ Yes ◯ No
Hard skin	◯ Yes ◯ No
Thick skin around nails	◯ Yes ◯ No
Deformation of nails	◯ Yes ◯ No
Loss of nails	◯ Yes ◯ No

LEFT FOOT: TOE 4

Live Fleas	__________________________
Dead Fleas	__________________________
Manipulated Lesions	__________________________
Cluster	__________________________
Peeling Skin	◯ Yes ◯ No
Cracks	◯ Yes ◯ No
Ulcers	◯ Yes ◯ No
Abscess	◯ Yes ◯ No
Hard skin	◯ Yes ◯ No
Thick skin around nails	◯ Yes ◯ No
Deformation of nails	◯ Yes ◯ No
Loss of nails	◯ Yes ◯ No

LEFT FOOT: TOE 5

Live Fleas	______________________
Dead Fleas	______________________
Manipulated Lesions	______________________
Cluster	______________________
Peeling Skin	◯ Yes ◯ No
Cracks	◯ Yes ◯ No
Ulcers	◯ Yes ◯ No
Abscess	◯ Yes ◯ No
Hard skin	◯ Yes ◯ No
Thick skin around nails	◯ Yes ◯ No
Deformation of nails	◯ Yes ◯ No
Loss of nails	◯ Yes ◯ No

LEFT FOOT: MEDIAL SIDE

Live Fleas	______________________
Dead Fleas	______________________
Manipulated Lesions	______________________
Cluster	______________________
Peeling Skin	◯ Yes ◯ No
Cracks	◯ Yes ◯ No
Ulcers	◯ Yes ◯ No
Abscess	◯ Yes ◯ No
Hard skin	◯ Yes ◯ No

LEFT FOOT: LATERAL SIDE

Live Fleas	__________________
Dead Fleas	__________________
Manipulated Lesions	__________________
Cluster	__________________
Peeling Skin	◯ Yes ◯ No
Cracks	◯ Yes ◯ No
Ulcers	◯ Yes ◯ No
Abscess	◯ Yes ◯ No
Hard skin	◯ Yes ◯ No

LEFT FOOT: HEEL

Live Fleas	__________________
Dead Fleas	__________________
Manipulated Lesions	__________________
Cluster	__________________
Peeling Skin	◯ Yes ◯ No
Cracks	◯ Yes ◯ No
Ulcers	◯ Yes ◯ No
Abscess	◯ Yes ◯ No
Hard skin	◯ Yes ◯ No

LEFT FOOT: SOLE

Live Fleas ______________________

Dead Fleas ______________________

Manipulated Lesions ______________________

Cluster ______________________

Peeling Skin ◯ Yes ◯ No

Ulcers ◯ Yes ◯ No

Cracks ◯ Yes ◯ No

Abscess ◯ Yes ◯ No

Hard skin ◯ Yes ◯ No

TOTAL FLEA COUNT

Total Flea count ______________________

Total Cluster count ______________________

Intensity of infection
◯ LOW
◯ MEDIUM
◯ HIGH

RIGHT FOOT: TOE 1

Infra-red white spots ◯ Yes ◯ No

RIGHT FOOT: TOE 2

Infra-red white spots ◯ Yes ◯ No

RIGHT FOOT : TOE 3

Infra-red white spots ◯ Yes ◯ No

RIGHT FOOT:TOE 4

Infra-red white spots ◯ Yes ◯ No

RIGHT FOOT: TOE 5

Infra-red white spots ◯ Yes ◯ No

RIGHT FOOT:MEDIAL SIDE

Infra-red white spots ◯ Yes ◯ No

RIGHT FOOT:LATERAL SIDE

Infra-red white spots ◯ Yes ◯ No

RIGHT FOOT: HEEL SIDE

Infra-red white spots ◯ Yes ◯ No

RIGHT FOOT:SOLE SIDE

Infra-red white spots ◯ Yes ◯ No

LEFT:Toe 1

Infra-red white spots ◯ Yes ◯ No

LEFT:Toe 2

Infra-red white spots ◯ Yes ◯ No

LEFT:Toe 3

Infra-red white spots ◯ Yes ◯ No

LEFT:Toe 4

Infra-red white spots ◯ Yes ◯ No

LEFT:Toe 5

Infra-red white spots ◯ Yes ◯ No

Left foot:Medial

Infra-red white spots ◯ Yes ◯ No

Left foot:Lateral

Infra-red white spots ◯ Yes ◯ No

Left Foot:Heel

Infra-red white spots ◯ Yes ◯ No

Left foot:Sole

Infra-red white spots ◯ Yes ◯ No

Other skin infections/conditions

Any other skin condition/infection apart from tungiasis?
◯ Yes
◯ No

If yes, which skin infection/condition?
☐ Scabies
☐ Ring worm
☐ Podoconiosis
☐ Cutaneous larva migrans
☐ Myiasis
☐ Warts
☐ Others (specify)

Specify (others) ______________________________

During the last week rate the following according to the scales

	Not at all	Only a little	Quite a lot	Very much
How much Itching do you feel?	◯	◯	◯	◯
How much pain do you feel ?	◯	◯	◯	◯

Total ______________________________

Any comments (if no leave blank)? ______________________________
