# Supplementary material for: Individual and household factors associated with tungiasis in a marginalized population in Karamoja, northeastern Uganda
Source: Trop Med Health. 2026 Mar 3;54:36. doi: 10.1186/s41182-025-00841-2 (PMC12954993; doi:10.1186/s41182-025-00841-2)
Supplement: Supplementary file 1 — Supplementary Material 1 [file 41182_2025_841_MOESM1_ESM.zip › Elson Karamoja tungiasis child foot exam.pdf]

Pupil/Child Screening Tool

ID	______________________
Date	______________________
Enumerator (Interviewer) Initials	______________________ (e.g. JM for Jane Mukisa)
Region	◯ BU<br>◯ SI<br>◯ KW<br>◯ NA
Sub county	◯ Ngoleriet<br>◯ Iriri<br>◯ Lokopo<br>◯ Lotome<br>◯ Matany<br>◯ Lopeei<br>◯ Lorengecora<br>◯ Matany T/C<br>◯ Kangole T/C<br>◯ Lorengecora (T/C)<br>◯ Other
Specify other sub county	______________________
Parish	______________________
Name of School or Village of index children recruitment	______________________
Recruitment School or Village ID number (from school or Village list)	______________________ (e.g. 200)
School or Village project ID (Region_School or Village ID no.)	______________________ (e.g. NA_200)
Name of Child (first and family name)	______________________ (e.g. John Doe)
Child's Home Village	______________________ (residence)

Child's Home Mannyatta	________________________________<br>(Indicate where "Not applicable' if the house is not located in a manyatta)
Child's Age	________________________________<br>(state age in years)
Does the child go to school?	◯ Yes<br>◯ No
Name of school the child is attending	________________________________
Class (including stream if any)	________________________________
Sex	◯ Male<br>◯ Female
Project child ID (pre-defined consecutive number 001-102)	________________________________<br>(e.g. 001)
MUAC (cm):	________________________________<br>(cm)
Weight (kg)	________________________________<br>(kg)
Height (cm)	________________________________
Is the FLOOR in the place YOU SLEEP made from	◯ mud/sand (dirt/natural)<br>◯ concrete
Jiggers on hands ?	◯ Yes ◯ No
Number of Sand fleas on hands	________________________________<br>(state the figure)
Jiggers infection on feet?	◯ Yes ◯ No

RIGHT FOOT: TOE 1

Live Fleas	______________________
Dead Fleas	______________________
Manipulated Lesions	______________________
Cluster(s)	______________________
Peeling Skin	◯ Yes ◯ No
Cracks	◯ Yes ◯ No
Ulcers	◯ Yes ◯ No
Abscess	◯ Yes ◯ No
Hard skin	◯ Yes ◯ No
Thick skin around nails	◯ Yes ◯ No
Deformation of nails	◯ Yes ◯ No
Loss of nails	◯ Yes ◯ No

RIGHT FOOT: TOE 2

Live Fleas	______________________
Dead Fleas	______________________
Manipulated Lesions	______________________
Cluster(s)	______________________
Peeling Skin	◯ Yes ◯ No
Cracks	◯ Yes ◯ No
Ulcers	◯ Yes ◯ No
Abscess	◯ Yes ◯ No
Hard skin	◯ Yes ◯ No

Thick skin around nails	◯ Yes ◯ No
Deformation of nails	◯ Yes ◯ No
Loss of nails	◯ Yes ◯ No

RIGHT FOOT: TOE 3

Live Fleas	__________________
Dead Fleas	__________________
Manipulated Lesions	__________________
Cluster(s)	__________________
Peeling Skin	◯ Yes ◯ No
Cracks	◯ Yes ◯ No
Ulcers	◯ Yes ◯ No
Abscess	◯ Yes ◯ No
Hard skin	◯ Yes ◯ No
Thick skin around nails	◯ Yes ◯ No
Deformation of nails	◯ Yes ◯ No
Loss of nails	◯ Yes ◯ No

RIGHT FOOT: TOE 4

Live Fleas	__________________
Dead Fleas	__________________
Manipulated Lesions	__________________
Cluster(s)	__________________
Peeling Skin	◯ Yes ◯ No

Cracks	◯ Yes ◯ No
Ulcers	◯ Yes ◯ No
Abscess	◯ Yes ◯ No
Hard skin	◯ Yes ◯ No
Thick skin around nails	◯ Yes ◯ No
Deformation of nails	◯ Yes ◯ No
Loss of nails	◯ Yes ◯ No

RIGHT FOOT: TOE 5

Live Fleas	__________________________
Dead Fleas	__________________________
Manipulated Lesions	__________________________
Cluster(s)	__________________________
Peeling Skin	◯ Yes ◯ No
Cracks	◯ Yes ◯ No
Ulcers	◯ Yes ◯ No
Abscess	◯ Yes ◯ No
Hard skin	◯ Yes ◯ No
Thick skin around nails	◯ Yes ◯ No
Deformation of nails	◯ Yes ◯ No
Loss of nails	◯ Yes ◯ No

RIGHT FOOT: MEDIAL SIDE

Live Fleas	______________________
Dead Fleas	______________________
Manipulated Lesions	______________________
Cluster(s)	______________________
Peeling Skin	◯ Yes ◯ No
Cracks	◯ Yes ◯ No
Ulcers	◯ Yes ◯ No
Abscess	◯ Yes ◯ No
Hard skin	◯ Yes ◯ No

RIGHT FOOT: LATERAL SIDE

Live Fleas	______________________
Dead Fleas	______________________
Manipulated Lesions	______________________
Cluster(s)	______________________
Peeling Skin	◯ Yes ◯ No
Cracks	◯ Yes ◯ No
Ulcers	◯ Yes ◯ No
Abscess	◯ Yes ◯ No
Hard skin	◯ Yes ◯ No

RIGHT FOOT: HEEL

Live Fleas	________________
Dead Fleas	________________
Manipulated Lesions	________________
Cluster(s)	________________
Peeling Skin	◯ Yes ◯ No
Cracks	◯ Yes ◯ No
Ulcers	◯ Yes ◯ No
Abscess	◯ Yes ◯ No
Hard skin	◯ Yes ◯ No

RIGHT FOOT: SOLE

Live Fleas	________________
Dead Fleas	________________
Manipulated Lesions	________________
Cluster(s)	________________
Peeling Skin	◯ Yes ◯ No
Cracks	◯ Yes ◯ No
Ulcers	◯ Yes ◯ No
Abscess	◯ Yes ◯ No
Hard skin	◯ Yes ◯ No

LEFT FOOT: TOE 1

Live Fleas	______________________
Dead Fleas	______________________
Manipulated Lesions	______________________
Cluster(s)	______________________
Peeling Skin	◯ Yes ◯ No
Cracks	◯ Yes ◯ No
Ulcers	◯ Yes ◯ No
Abscess	◯ Yes ◯ No
Hard skin	◯ Yes ◯ No
Thick skin around nails	◯ Yes ◯ No
Deformation of nails	◯ Yes ◯ No
Loss of nails	◯ Yes ◯ No

LEFT FOOT: TOE 2

Live Fleas	______________________
Dead Fleas	______________________
Manipulated Lesions	______________________
Cluster(s)	______________________
Peeling Skin	◯ Yes ◯ No
Cracks	◯ Yes ◯ No
Ulcers	◯ Yes ◯ No
Abscess	◯ Yes ◯ No
Hard skin	◯ Yes ◯ No

Thick skin around nails	◯ Yes ◯ No
Deformation of nails	◯ Yes ◯ No
Loss of nails	◯ Yes ◯ No

LEFT FOOT: TOE 3

Live Fleas	__________________________
Dead Fleas	__________________________
Manipulated Lesions	__________________________
Cluster(s)	__________________________
Peeling Skin	◯ Yes ◯ No
Cracks	◯ Yes ◯ No
Ulcers	◯ Yes ◯ No
Abscess	◯ Yes ◯ No
Hard skin	◯ Yes ◯ No
Thick skin around nails	◯ Yes ◯ No
Deformation of nails	◯ Yes ◯ No
Loss of nails	◯ Yes ◯ No

LEFT FOOT: TOE 4

Live Fleas	__________________________
Dead Fleas	__________________________
Manipulated Lesions	__________________________
Cluster(s)	__________________________
Peeling Skin	◯ Yes ◯ No

Cracks	◯ Yes ◯ No
Ulcers	◯ Yes ◯ No
Abscess	◯ Yes ◯ No
Hard skin	◯ Yes ◯ No
Thick skin around nails	◯ Yes ◯ No
Deformation of nails	◯ Yes ◯ No
Loss of nails	◯ Yes ◯ No

LEFT FOOT: TOE 5

Live Fleas	________________
Dead Fleas	________________
Manipulated Lesions	________________
Cluster(s)	________________
Peeling Skin	◯ Yes ◯ No
Cracks	◯ Yes ◯ No
Ulcers	◯ Yes ◯ No
Abscess	◯ Yes ◯ No
Hard skin	◯ Yes ◯ No
Thick skin around nails	◯ Yes ◯ No
Deformation of nails	◯ Yes ◯ No
Loss of nails	◯ Yes ◯ No

LEFT FOOT: MEDIAL SIDE

Live Fleas	________________
Dead Fleas	________________
Manipulated Lesions	________________
Cluster(s)	________________
Cracks	◯ Yes ◯ No
Peeling Skin	◯ Yes ◯ No
Ulcers	◯ Yes ◯ No
Abscess	◯ Yes ◯ No
Hard skin	◯ Yes ◯ No

LEFT FOOT: LATERAL SIDE

Live Fleas	________________
Dead Fleas	________________
Manipulated Lesions	________________
Cluster(s)	________________
Peeling Skin	◯ Yes ◯ No
Cracks	◯ Yes ◯ No
Ulcers	◯ Yes ◯ No
Abscess	◯ Yes ◯ No
Hard skin	◯ Yes ◯ No

LEFT FOOT: HEEL

Live Fleas	______________
Dead Fleas	______________
Manipulated Lesions	______________
Cluster(s)	______________
Peeling Skin	◯ Yes ◯ No
Cracks	◯ Yes ◯ No
Ulcers	◯ Yes ◯ No
Abscess	◯ Yes ◯ No
Hard skin	◯ Yes ◯ No

LEFT FOOT: SOLE

Live Fleas	______________
Dead Fleas	______________
Manipulated Lesions	______________
Cluster(s)	______________
Peeling Skin	◯ Yes ◯ No
Cracks	◯ Yes ◯ No
Ulcers	◯ Yes ◯ No
Abscess	◯ Yes ◯ No
Hard skin	◯ Yes ◯ No

RIGHT FOOT:

toe 1

Infra-red white spots toe 1
◯ Yes
◯ No

toe 2

Infra-red white spots toe 2
◯ Yes
◯ No

toe 3

Infra-red white spots toe 3
◯ Yes
◯ No

toe 4

Infra-red white spots toe 4
◯ Yes
◯ No

toe 5

Infra-red white spots toe 5
◯ Yes
◯ No

Medial side

Infra-red white spots medial side
◯ Yes
◯ No

Lateral side

Infra-red white spots lateral side
◯ Yes
◯ No

Heel

Infra-red white spots heel side
◯ Yes
◯ No

sole

Infra-red white spots sole
◯ Yes
◯ No

LEFT FOOT :

toe 1

Infra-red white spots left toe 1

◯ Yes
◯ No

toe 2

Infra-red white spots left toe 2

◯ Yes
◯ No

toe 3

Infra-red white spots left toe 3

◯ Yes
◯ No

toe 4

Infra-red white spots left toe 4

◯ Yes
◯ No

toe 5

Infra-red white spots left toe 5

◯ Yes
◯ No

Medial side

Infra-red white spots left medial side

◯ Yes
◯ No

Lateral side

Infra-red white spots left lateral side

◯ Yes
◯ No

Heel side

Infra-red white spots left heel side

◯ Yes
◯ No

sole side

Infra-red white spots left sole side

◯ Yes
◯ No

Total lesion count for the child

____

Total cluster count for the child

____

0-5 LOW

6-30 : MEDIUM

>30 : HIGH

cluster count

>1 :HIGH

Indicate intensity (based on scale above):	◯ LOW<br>◯ MEDIUM<br>◯ HIGH
Other skin infection/condition observed	◯ Yes<br>◯ No
Name other skin infection/condition	☐ Scabies<br>☐ Ringworm<br>☐ Podoconiosis<br>☐ Cutaneous larva migrans<br>☐ Myiasis<br>☐ Warts<br>☐ Other (specify)
Specify the other skin infection/condition	______________________
Ask the child if anyone else is infected in household?:	◯ Yes<br>◯ No
SELECTED AS INDEX CHILD	◯ No<br>◯ Yes<br>◯ Stand-by
Selected for IMPACT study?	◯ No<br>◯ Yes<br>◯ Stand-by

During the last week rate the following according to the scales

	Not at all	Only a little	Quite a lot	Very much
How much pain due to jiggers did you feel?	◯	◯	◯	◯
How much Itching due to jiggers did you feel?	◯	◯	◯	◯

Total	______________________
Any further comments (leave blank if none)?	______________________<br>(ADD COMMENTS IF THERE IS SOMETHING YOU FOUND OD[illegible]<br>OR UNIQUE)
