# Supplementary material for: Individual and household factors associated with tungiasis in a marginalized population in Karamoja, northeastern Uganda
Source: Trop Med Health. 2026 Mar 3;54:36. doi: 10.1186/s41182-025-00841-2 (PMC12954993; doi:10.1186/s41182-025-00841-2)
Supplement: Supplementary file 1 — Supplementary Material 1 [file 41182_2025_841_MOESM1_ESM.zip › Elson Karamoja tungiasis_HH_Questionnaire.docx]

Household Survey

*Page 1*

Household ID number

Date

Sub County

Ngoriet

Iriri

Lokopo

Lotome

Matany

Lopeei

Lorengecora

Matany T/C

Kangole T/C

Lorengecora

Others

(

where the village is located

)

School/Village ID number

from where the index child was selected

(e.g 093)

Index child project ID

(e.g. KW_093_001)

Name of index child

(First and family name)

latitude

longitude

Home village

Name of head of household:

Sex of head of household: male

female


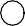

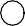


Age of head of household

Name of care giver:

Sex of care giver: Male

Female


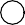

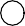


Age of care giver:

Care givers relation to index child? parent grandparent sibling aunt/uncle cousin


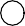

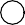

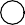

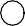

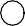

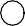


other (specify)

If other relation, what kind?

**Socioeconomic factors**

Do you (your family) own this land on which you have Yes its owned, your house? no its rented,


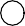

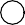

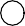

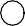


no we are sqatters, no-other reasons.

Other reasons

Does this family own ADDITIONAL land for farming? Yes No

**Select Yes if the family owns any below or NO if not**

Yes No


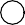

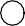


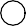

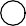

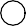

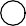
radio tv


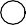

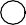

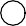

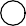
mobile phone bicycle


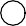

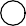

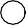

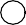
motor cycle solar system


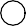

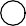
car, tractor or boat

4. Do you make/ brew any alcohol from this home?

Yes

No (observe)


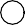

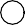


**Water, Sanitation and Hygiene**

What is your PRIMARY (most often used) source of tap in compound


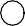

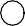

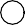

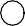

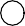

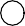

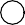


water? shared community tap

own well

shared community well or borehole river or lake or pond

rainwater collection in tank/drums combination - specify

specify water source

Does any of the following apply? (if yes, select,
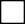
 The household pays for the daily water supply. multiple answers possible)
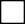
 Some or all of the water needed is brought by a

hired motorbike.


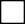
 Some or all of the water needed is brought by a hired donkey cart.


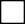
 Some or all of the water needed is fetched by household members from a short distance.


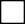
 Some or all of the water needed is fetched by household members from a long distance.


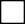
 None of above applies. (read the options )

How many jerry-cans (of large 20 L type) of water do

you use in a day for BATHING (all family members

total)? (Not including water for cooking, drinking or

laundry)

In which location do you bathe most of the time? in river/pond/lake,

with basin of water anywhere on compound/in the bushes/garden nearby


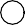

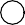


built bathroom/latrine building makeshift bathroom


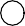

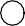

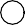


other bathing place

Other bathing place

How often do you bathe your feet? twice a day once a day not every day

Do you use soap when you bathe your feet? no sometimes always


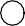

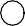

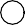


Where do you go (most of the time) to defecate? flush toilet ventilated pit latrine traditional latrine bush

How does your family dispose waste most of the time? open/roadside/in the garden/bush/farm

collected in pit collected and burned other - specify


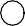

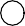

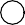

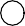

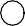

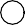

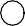

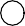


Other waste disposal

**Nutrition**

How many meals did the family eat yesterday?

**Caregiver behaviour (focus on the index child)**

What is the relationship of [name_of_index_child] to Child,


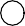

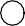

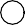

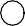

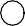


household head? Grandchild,

Niece/nephew, Adopted orphan Other-specify

Other relation

Does [name_of_index_child] have any disabilities ? Physical, mental, Both, None.

Does [name_of_index_child] have any current illnesses?
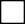
 none


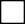
 Respiratory, chest, cough, nose
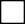
 diarrhea, stomach


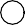

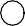

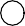

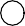


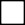
 skin rashes,
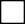
 eye problems
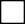
 ear problems
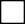
 headache


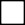
 Fever, malaria
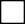
 Others

index child other illness

Does the index child have a chronic (meaning Yes

long-term) illness? No


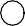

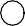


If the child has a chronic (long term) illnesses, what
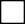
 HIV

kind?
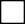
 Cancer


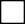
 Diabetes
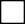
 Asthma


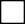
 Cystic fibrosis (affects lungs and digestive system)


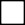
 Heart disease/defect


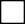
 Other organ (not heart) defects
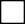
 Recurring diarrhoea/blood in stool
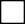
 DONT KNOW or not diagnosed (e.g. asthma, cancer, diabetes)

Did you/the mother have any birth complications with no

[name_of_index_child]? yes


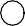

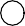

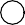


don't know


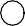

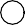


How often do you yourself (not somebody else) bathe twice a day, the younger children? once a day,

not every day never


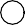

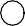

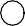

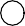


At around what age of the children do you stop bathing/washing them yourself?

Do you or anyone else help or supervise no

[name_of_index_child] bathe? yes


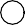


don't know

If yes, who?

(e.g. self, sibling, house help, etc)

How often does [name_of_index_child] bathe? twice a day or more, once a day,

not every day never

don't know

Does [name_of_index_child] use soap for bathing? never sometimes always don't know

How much time do you spend talking with None

[name_of_index_child] each day? a little some a lot

Do you hug/cuddle [name_of_index_child]? Yes No

If yes, how often? Several times a day

once a day not every day

Do you think you did hug and cuddle Yes

[name_of_index_child] more often when he/she was No younger?

Did you spend more or less time with the child when more

he/she was younger? less

the same don't know

What did you do the last time [name_of_index_child] nothing

did something wrong? beat her/him,

talk to her/him calmly about mistake, send away/sit in one place,

shout at him/her other -specify

(Do not read out the reason only select based on what the respondent says)

Other

Do you know the friends of [name_of_index_child] ? Yes No

Do you know the parents of your child's friends? Yes No

**Homestead and household characteristics**

How many adults (18 and above) live in your household including yourself?

How many children below the age or 5 live in your household?

How many children between 6 and 17 life in your household including the index child?

Is your household part of a larger homestead with Yes

other households? No

If yes, how many other family households are in the homestead?

How is the index child related to the other grandchild

households' heads? niece/nephew

other - specify

grandchild and niece/nephew

other relation

In total, how many adults (18 and above) live IN THE OTHER households?

In total, how many children (less than 18 years) live IN THE OTHER households?

Do you (the index household) share any resources Receive support with the other households in your homestead (e.g. Give support

provide or receive financial support, sharing of Mutual

motorbike, provide or receive food etc? don't share any resources

What kind of support do you receive?

Does anyone in the other households have jiggers? Yes

No

How many of the other households are affected?

How many children are affected in all the other households together?

How many adults are affected in all the other households together?

**Request to be shown the INDEX household (support your questions with observations**

How many structures belong to the household? main house, kitchen house,

teenage boys' hut/small house/simba latrine/shower

other - specify

Other structures

How many sleeping areas are in the main house?

Specify the areas Adults only

Mixed adults & children bedroom

Children only bedroom (mixed boys/girls) Boys bedroom

Girls bedroom

Grandparent bedroom

Grandparent bedroom shared with (some) children Children in lounge

Other(specify)

(example: parent's bedroom children living room floor, etc)

How many adults sleep in the main house?

(if none enter 0)

How many children sleep in main house (but not in same bed as mother)

Observe the wall material of main house permanent stone or brick

semi-permanent mud/cement or mud/stone mudpalm leaves/grass/matt other-specify

other wall material

Observe the roof material of main house tiles

iron sheets

thatch (dry vegetation e.g grass, palm leaves etc) other-specify

Other roof material main house

Observe the state of repair of main house good

poor

very poor (holes in walls & roof)

State of sanitation inside the main house swept & tidy,

not swept, items scattered throughout other (specify

Other sanitation main house

**Sleeping conditions of index child. Request to be shown where the index child sleeps**

In which structure does the index child sleep? Adults only

Mixed adults & children bedroom Children only bedroom (mixed boys/girls) Boys bedroom

Girls bedroom Grandparent bedroom

Grandparent bedroom shared with (some) children Children in lounge

Other(specify)

other sleeping place for index child

Does the index child share the sleeping place Yes

with other children? No

Observe (ask if unclear). Does the child sleep on Yes

a raised bed/structure? No

On what surface does the child sleep? mattress

rugs/old sheets/mosquito nets reed mats

nothing (bare frame or floor)

How tidy is the sleeping place of index child? swept & tidy,

not swept, items scattered throughout other (specify

other state of sleeping place

Is there any organic material in the same room Yes

(e.g. maize, beans, flour etc) No
