# Supplementary material for: Individual and household factors associated with tungiasis in a marginalized population in Karamoja, northeastern Uganda
Source: Trop Med Health. 2026 Mar 3;54:36. doi: 10.1186/s41182-025-00841-2 (PMC12954993; doi:10.1186/s41182-025-00841-2)
Supplement: Supplementary file 2 — Supplementary Material 2. [file 41182_2025_841_MOESM2_ESM.docx]

**Additional file 2**

**High density rural settlements, parenting behavior and child disability are significant factors associated with tungiasis in Karamoja, northeastern Uganda: a case control study.**

Lynne Elson^1,2^, Abneel K. Matharu^3,4^, Berrick Otieno^5^, Herman Feldmeier^6^, Charles Waiswa^7^, Amina Abubakar^5^, Jürgen Krücken^3,8^, Ulrike Fillinger^4,5^, Francis Mutebi^6 *^

^1^ KEMRI-Wellcome Trust Research Programme, Kilifi, Kenya

^2^ Centre for Tropical Medicine and Global Health, Nuffield Department of Medicine, University of Oxford, Oxford, United Kingdom.

^3^ Institute for Parasitology and Tropical Veterinary Medicine, Freie Universität Berlin, Berlin, Germany

^4^ International Centre of Insect Physiology and Ecology (ICIPE), Human Health Theme, Nairobi, Kenya

^5^ Institute for Human Development, Aga Khan University, Nairobi, Kenya

^6^ Institute of Microbiology, Infectious Diseases and Immunology, Charité University Medicine, Berlin, Germany

^7^ College of Veterinary Medicine, Animal Resources and Biosecurity, Makerere University, Kampala, Uganda

^8^ Veterinary Centre for Resistance Research, Freie Universität Berlin, Berlin, Germany

***Correspondence**

[francmutebi10@gmail.com](mailto:francmutebi10@gmail.com)

Contents

[S1: Stata output results for the polychoric factor analysis for Socioeconomic status 3](#_Toc203746313)

[Table S2: Infection intensity score of all cases by age groups and sex 6](#_Toc203746314)

[Table S3: Household characteristics 6](#_Toc203746315)

[Table S4: Univariable logistic regression for household risk factors for tungiasis, village as random effect 11](#_Toc203746316)

[Table S5: Adult and Household factors associated with socioeconomic status 15](#_Toc203746317)

## S1: Stata output results for the polychoric factor analysis for Socioeconomic status

Variables included ownership of assets: radio, mobile phone, bicycle, motorcycle, solar power unit, livestock.

**Polychoric correlation matrix**

|  | Radio | Mobile phone | Bicycle | Motorcycle | Solar | Livestock |
| --- | --- | --- | --- | --- | --- | --- |
| Radio | 1 |  |  |  |  |  |
| Mobile phone | 0.659 | 1 |  |  |  |  |
| Bicycle | 0.351 | 0.456 | 1 |  |  |  |
| Motorcycle | 0.481 | 0.404 | 0.357 | 1 |  |  |
| Solar | 0.546 | 0.582 | 0.503 | 0.250 | 1 |  |
| Livestock | 0.322 | 0.361 | 0.574 | 0.144 | 0.239 | 1 |

**Factor matrix for eigen values**

Factor analysis/correlation Number of obs = 568

Method: principal factors Retained factors = 3

Rotation: (unrotated) Number of params = 15

| Factor | Eigenvalue | Difference | Proportion | Cumulative |
| --- | --- | --- | --- | --- |
| Factor1 | 2.60831 | 2.16665 | 0.9291 | 0.9291 |
| Factor2 | 0.44166 | 0.29763 | 0.1573 | 1.0864 |
| Factor3 | 0.14403 | 0.11640 | 0.0513 | 1.1378 |
| Factor4 | 0.02762 | 0.15510 | 0.0098 | 1.1476 |
| Factor5 | -0.12747 | 0.15939 | -0.0454 | 1.1022 |
| Factor6 | -0.28686 | . | -0.1022 | 1.0000 |

LR test: independent vs. saturated: chi2(15) = 1268.87 Prob>chi2 = 0.0000

**Factor loadings (pattern matrix) and unique variances**

| Variable | Factor1 | Factor2 | Factor3 | Uniqueness |
| --- | --- | --- | --- | --- |
| Radio | 0.7435 | -0.2695 | 0.0481 | 0.3722 |
| Mobile phone | 0.7688 | -0.1484 | -0.0505 | 0.3844 |
| Bicycle | 0.6882 | 0.3622 | 0.0199 | 0.3948 |
| Motorcycle | 0.5060 | -0.1782 | 0.2668 | 0.6410 |
| Solar | 0.6829 | -0.0907 | -0.2543 | 0.4608 |
| livestock | 0.5181 | 0.4194 | 0.0542 | 0.5527 |

**Scree plot of eigen values for each factor**

**Loading plot for factor 1 and 2**

**Scoring coefficients (method = regression)**

| Variable | Factor1 | Factor2 | Factor3 |
| --- | --- | --- | --- |
| Radio | 0.288 | -0.314 | 0.134 |
| Mobile phone | 0.269 | -0.157 | -0.073 |
| Bicycle | 0.273 | 0.430 | 0.079 |
| Motorcycle | 0.100 | -0.147 | 0.300 |
| Solar | 0.180 | -0.096 | -0.410 |
| Livestock | 0.115 | 0.375 | 0.047 |

(variable means assumed 0; use means() option of factormat for nonzero means)

(variable std. deviations assumed 1; use sds() option of factormat to change)

**Kaiser-Meyer-Olkin measure of sampling adequacy for factor 1**

| Variable | kmo |
| --- | --- |
| Radio | 0.7246 |
| Mobile phone | 0.8229 |
| Bicycle | 0.6438 |
| Motorcycle | 0.6694 |
| Solar | 0.7085 |
| Livestock | 0.6001 |
| **Overall** | **0.7021** |

Frequency distribution of socioeconomic factor 1

## Table S2: Association of socioeconomic status with adult and household factors

Bivariable mixed effect linear regression for socioeconomic status, village identifier as random effects

| **Variable** | **Categories** | **N^a^** | **Coefficient** | **95% CI^b^** | | **P^c^** |
| --- | --- | --- | --- | --- | --- | --- |
| Adult age group | 18-24 | 95 | 0 |  |  |  |
|  | 25-44 | 352 | -0.05 | -0.11 | 0.01 | 0.094 |
|  | 45-60 | 160 | -0.06 | -0.13 | -0.01 | 0.064 |
|  | >60 | 86 | -0.12 | -0.20 | -0.04 | 0.002 |
| Adult Marital status | Married | 540 | 0 |  |  |  |
|  | Other | 153 | -0.13 | -0.17 | -0.08 | <0.001 |
| Adult Education level | None | 474 | 0 |  |  |  |
|  | Some primary | 151 | 0.12 | 0.07 | 0.16 | <0.001 |
|  | More | 68 | 0.17 | 0.10 | 0.23 | <0.001 |
| Adult occupation | Selling goods | 76 | 0 |  |  |  |
|  | None | 74 | -0.20 | -0.29 | -0.11 | <0.001 |
|  | Casual labor | 367 | -0.15 | -0.21 | -0.09 | <0.001 |
|  | Farming | 32 | 0.03 | -0.08 | 0.14 | 0.553 |
|  | Other | 13 | 0.04 | -0.10 | 0.18 | 0.592 |
| HH^d^ in a manyatta | No | 200 | 0 |  |  |  |
|  | Yes | 368 | -0.16 | -0.24 | -0.09 | <0.001 |
| Brew alcohol at home | No | 366 | 0 |  |  |  |
|  | Yes | 202 | 0.16 | 0.11 | 0.20 | <0.001 |

^a^ number of households, ^b^ confidence interval, ^c^ associated p-value, ^d^ household

## Table S2: Infection intensity score of all cases by age groups and sex

|  | All | | | Female | | | Male | | |  |
| --- | --- | --- | --- | --- | --- | --- | --- | --- | --- | --- |
| Age group (years) | N^a^ | Median, (IQR^b^) | % severe^c^ | N | Median (IQR) | % severe | N | Median (IQR) | % severe | P-value^d^ |
| 0-7 | 161 | 7 (3-16) | 39.1 | 82 | 7 (3-19) | 40.2 | 79 | 8 (3-14) | 38.0 | 0.754 |
| 8-14 | 929 | 8 (3-20) | 40.1 | 459 | 6 (3-18) | 35.9 | 470 | 9 (4-21) | 44.2 | 0.004 |
| 15-24 | 15 | 4 (3-12) | 26.7 | 11 | 4 (3-6) | 18.2 | 4 | 9 (3-98) | 50.0 | 0.402 |
| 25-44 | 84 | 6 (3-22) | 40.5 | 79 | 7 (3-22) | 41.8 | 5 | 4 (2-26) | 20.0 | 0.504 |
| 45-60 | 68 | 11 (3-41) | 50.0 | 54 | 12 (3-35) | 53.7 | 14 | 6 (3-54) | 35.7 | 0.401 |
| >60 | 41 | 18 (5-38) | 63.4 | 33 | 18 (7-39) | 66.7 | 8 | 17 (3-34) | 50.0 | 0.790 |
| Total | 1298 | 8 (3-20) | 41.4 | 718 | 7 (3-21) | 39.6 | 580 | 9 (4-20) | 43.0 | 0.083 |

^a^ Number of cases, ^b^ Interquartile range, ^c^ % of cases with >10 fleas, ^d^p value from Wilcoxon test for difference in intensity score between sexes

## Table S3: Household characteristics by infection status

| **Variable** | **Missing** | **Category** | **All** | **Controls** | **Cases** | **p-value ^a^** |
| --- | --- | --- | --- | --- | --- | --- |
| All |  |  | 568 | 358 | 210 (37.1) |  |
| HH^b^ in a manyatta |  | No | 200 | 190 | 10 (5.0) | <0.001 |
|  |  | Yes | 368 | 168 | 200 (54.4) |  |
| Sex index child | 0 | Male | 230 | 127 | 103 (44.8) | 0.002 |
|  |  | Female | 339 | 231 | 108 (31.9) |  |
| Sex HH head | 0 | Male | 54 | 41 | 13 (24.1) | 0.038 |
|  |  | Female | 515 | 317 | 198 (38.5) |  |
| Sex caregiver | 0 | Male | 32 | 19 | 13 (40.6) | 0.669 |
|  |  | Female | 537 | 339 | 198 (36.9) |  |
| Adult over 60 years in HH | 0 | No | 502 | 325 | 177 (35.3) | 0.020 |
|  |  | Yes | 66 | 33 | 33 (50.0) |  |
| Caregiver relation to index | 0 | Parent | 404 | 262 | 142 (35.2) | 0.135 |
|  |  | Other | 165 | 96 | 69 (41.8) |  |
| Land status | 0 | Not owned | 185 | 114 | 71 (38.4) | 0.657 |
|  |  | Owned | 384 | 244 | 140 (36.5) |  |
| Own farmland | 4 | No | 174 | 121 | 53 (30.5) | 0.026 |
|  |  | Yes | 390 | 233 | 157 (40.3 |  |
| Water source | 3 | Piped or own well | 31 | 30 | 1 (3.2) | 0.000 |
|  |  | Shared borehole | 415 | 216 | 199 (48.0) |  |
|  |  | Unimproved/open | 119 | 110 | 9 (7.6) |  |
| Pay for water | 0 | No | 559 | 353 | 206 (36.9) | 0.394 |
|  |  | Yes | 10 | 5 | 5 (50.0) |  |
| Collect water from short distance | 0 | No | 251 | 172 | 79 (31.5) | 0.014 |
|  |  | Yes | 318 | 186 | 132 (41.5) |  |
| Collect water from long distance | 0 | No | 307 | 182 | 125 (40.7) | 0.052 |
|  |  | Yes | 262 | 176 | 86 (32.8) |  |
| Jerricans water used/day | 3 | <1.5 | 154 | 101 | 53 (34.4) | 0.180 |
|  |  | 1.5-3 | 324 | 194 | 130 (40.1) |  |
|  |  | >3 | 87 | 60 | 27 (31.0) |  |
| Bathe place | 5 | Built bathroom | 38 | 20 | 18 (47.4) | 0.043 |
|  |  | Basin on compound | 378 | 230 | 148 (39.2) |  |
|  |  | Makeshift bathroom | 147 | 104 | 43 (29.3) |  |
| Caregiver foot wash frequency | 3 | Twice a day | 344 | 227 | 117 (34.0) | 0.070 |
|  |  | Less often | 224 | 131 | 93 (41.5) |  |
| Caregiver soap use | 1 | Not always | 513 | 316 | 197 (38.4) | 0.038 |
|  |  | Always | 54 | 41 | 13 (24.1) |  |
| Toilet | 1 | latrine | 111 | 71 | 40 (36.0) | 0.808 |
|  |  | Open defecation | 456 | 286 | 170 (37.3) |  |
| Waste disposal | 6 | Discarded anywhere | 377 | 236 | 141 (37.4) | 0.471 |
|  |  | In a pit | 55 | 39 | 16 (29.1) |  |
|  |  | Collected and burned | 130 | 81 | 49 (37.7) |  |
| Number of meals yesterday | 0 | 0 or 1 | 383 | 219 | 164 (42.8) | 0.000 |
|  |  | 2 or 3 | 185 | 139 | 47 (24.9) |  |
| Grow own food | 15 | No | 29 | 13 | 16 (55.2) | 0.038 |
|  |  | Yes | 524 | 335 | 189 (36.1) |  |
| Brew alcohol at home | 0 | No | 366 | 225 | 141 (38.5) | 0.302 |
|  |  | Yes | 202 | 133 | 69 (34.2) |  |
| Index relation to HHH | 0 | Child | 432 | 276 | 156 (36.1) | 0.426 |
|  |  | Grandchild | 63 | 35 | 28 (44.4) |  |
|  |  | Other | 73 | 47 | 26 (35.6) |  |
| Index disability | 0 | No | 532 | 347 | 185 (34.8) | 0.000 |
|  |  | Yes | 36 | 11 | 25 (69.4) |  |
| Index other current illness | 0 | Yes | 125 | 64 | 61 (48.8) | 0.002 |
|  |  | No | 444 | 294 | 150 (33.8) |  |
| Index respiratory illness | 0 | No | 494 | 319 | 175 (35.4) | 0.036 |
|  |  | Yes | 75 | 39 | 36 (48.0) |  |
| Index child wasted | 3 | No | 411 | 254 | 157 (38.2) | 0.157 |
| Weight-for-age |  | Moderate | 115 | 81 | 34 (29.6) |  |
|  |  | Severe | 39 | 22 | 17 (43.6) |  |
| Index child stunted | 4 | No | 482 | 301 | 181 (37.6) | 0.201 |
| Height-for-age |  | Moderate | 65 | 47 | 18 (27.7) |  |
|  |  | Severe | 17 | 9 | 8 (47.1) |  |
| Frequency wash young children | 2 | Twice a day | 329 | 214 | 115 (35.0) | 0.000 |
|  |  | Once a day | 121 | 88 | 33 (27.3) |  |
|  |  | Less often | 64 | 31 | 33 (51.6) |  |
|  |  | Never | 52 | 23 | 29 (55.8) |  |
| Age stop bathing children (years) | 0 | <5 | 88 | 50 | 38 (43.2) | 0.254 |
|  |  | 6-10 | 403 | 254 | 149 (37.0) |  |
|  |  | 11-15 | 78 | 54 | 24 (30.8) |  |
| Index child bathing supervised | 8 | No | 363 | 257 | 106 (29.2) | 0.000 |
|  |  | Yes | 197 | 98 | 99 (50.3) |  |
| Index child frequency wash feet | 0 | Twice a day | 388 | 235 | 153 (39.4) | 0.165 |
|  |  | Less often | 159 | 110 | 49 (30.8) |  |
|  |  | Don’t know | 21 | 13 | 8 (38.1) |  |
| Index child soap use | 0 | Not always | 521 | 322 | 199 (38.2) | 0.038 |
|  |  | Always | 28 | 24 | 4 (14.3) |  |
|  |  | Don’t know | 19 | 12 | 7 (36.8) |  |
| Time caregivers spent talking with index child | 0 | None/a little | 366 | 261 | 105 (28.7) | 0.000 |
|  |  | Some/ a lot | 203 | 97 | 106 (52.2) |  |
| Caregiver hugs index child | 3 | No | 278 | 149 | 129 (46.4) | 0.000 |
|  |  | Yes | 287 | 207 | 80 (27.9) |  |
| If yes, how often hug index child | 281 | >Once a day | 90 | 73 | 17 (18.9) | 0.060 |
|  |  | Once a day | 45 | 29 | 16 (35.6) |  |
|  |  | Not every day | 152 | 105 | 47 (30.9) |  |
| Hug index child more when younger | 0 | No | 172 | 79 | 93 (54.1) | 0.000 |
|  |  | Yes | 396 | 279 | 117 (29.6) |  |
| Discipline style | 0 | Beat/ shout | 316 | 189 | 127 (40.2) | 0.075 |
|  |  | Other | 252 | 169 | 83 (32.9) |  |
| Know index child’s friends | 1 | No | 271 | 185 | 86 (31.7) | 0.015 |
|  |  | Yes | 296 | 173 | 123 (41.6) |  |
| Know parents of index child’s friends | 0 | No | 298 | 199 | 99 (33.2) | 0.052 |
|  |  | Yes | 270 | 159 | 111 (41.1) |  |
| **Caregiver/head of household** |  |  |  |  |  |  |
| Education | 0 | None | 397 | 238 | 159 (40.1) | 0.026 |
|  |  | Some | 172 | 120 | 52 (30.2) |  |
| Marital status | 0 | Married | 447 | 292 | 155 (34.7) | 0.023 |
|  |  | Not married | 122 | 66 | 56 (45.9) |  |
| Occupation | 5 | Employed, other | 14 | 11 | 2 (15.4) | 0.002 |
|  |  | Casual labor | 367 | 219 | 148 (40.3) |  |
|  |  | Farming (crops or livestock) | 32 | 25 | 7 (21.9) |  |
|  |  | Selling goods (food, alcohol etc) | 76 | 59 | 17 (22.4) |  |
|  |  | None | 74 | 40 | 34 (46.0) |  |
| Disability | 6 | No | 519 | 331 | 188 (36.2) | 0.179 |
|  |  | Yes | 43 | 23 | 20 (46.5) |  |
| Alcohol use | 6 | No | 78 | 52 | 26 (33.3) | 0.469 |
|  |  | Yes | 484 | 302 | 182 (37.6) |  |
| **Household structure** |  |  |  |  |  |  |
| HH is in a shared homestead | 0 | No | 389 | 256 | 133 (34.2) | 0.042 |
|  |  | Yes | 179 | 106 | 77 (43.0) |  |
| If yes, other HH have jiggers | 390 | No | 106 | 87 | 19 (17.9) | 0.000 |
|  |  | Yes | 72 | 14 | 58 (80.6) |  |
| Had a separate kitchen hut | 0 | No | 469 | 292 | 177 (37.7) | 0.482 |
|  |  | Yes | 100 | 66 | 34 (34.0) |  |
| Had a separate teenager sleeping hut | 0 | No | 506 | 323 | 183 (36.2) | 0.200 |
|  |  | Yes | 63 | 35 | 28 (44.4) |  |
| Number adults (>18) | 0 | 1-2 | 497 | 318 | 179 (36.0) | 0.212 |
|  |  | >2 | 71 | 40 | 31 (43.7) |  |
| Number under 5 yrs | 0 | 0 | 153 | 89 | 64 (41.8) | 0.107 |
|  |  | 1-2 | 349 | 220 | 129 (37.0) |  |
|  |  | >2 | 67 | 49 | 18 (26.9) |  |
| Number 6-17 yrs | 0 | 0-2 | 359 | 239 | 120 (33.4) | 0.018 |
|  |  | >2 | 210 | 119 | 91 (43.3) |  |
| Number sleep rooms in main house | 8 | 1 | 523 | 328 | 195 (37.3) | 0.624 |
|  |  | 2 | 33 | 23 | 10 (30.3) |  |
|  |  | 3 | 4 | 2 | 2 (50.0) |  |
| Number children sleep in main house | 7 | 0 | 74 | 61 | 13 (17.6) | 0.001 |
|  |  | 1-2 | 257 | 158 | 99 (38.5) |  |
|  |  | >2 | 230 | 134 | 96 (41.7) |  |
| Wall main house | 8 | Sticks, leaves, grass | 411 | 243 | 168 (40.9) | 0.008 |
|  |  | Mud/mixed mud & stone | 147 | 108 | 39 (26.5) |  |
|  |  | Stone/ bricks /other | 2 | 1 | 1 |  |
| Roof of main house | 9 | Iron sheets | 81 | 61 | 20 (24.7) | 0.014 |
|  |  | Thatch of grass | 478 | 292 | 186 (38.9) |  |
| State of repair main house | 7 | Good | 373 | 258 | 115 (30.8) | 0.000 |
|  |  | Poor | 155 | 80 | 75 (48.4) |  |
|  |  | Very poor (holes in walls & roof) | 33 | 15 | 18 (54.6) |  |
| Sanitation main house | 7 | Swept & tidy | 287 | 203 | 84 (29.3) | 0.000 |
|  |  | Not swept, items scattered | 257 | 137 | 120 (46.7) |  |
|  |  | Other | 17 | 13 | 4 (23.5) |  |
| Place index child sleeps | 0 | Adults & children mixed | 424 | 265 | 159 (37.5) | 0.771 |
|  |  | Children only | 34 | 23 | 11 (32.4) |  |
|  |  | With grandparents | 21 | 11 | 10 (47.6) |  |
|  |  | Teenagers hut | 66 | 44 | 22 (33.3) |  |
|  |  | Another hut | 23 | 15 | 8 (34.8) |  |
| Floor in index child sleep room | 6 | Hard clay | 398 | 282 | 116 (29.2) | 0.000 |
|  |  | Loose clay/soil | 155 | 64 | 91 (58.7) |  |
|  |  | Other | 15 | 12 | 3 (20.0) |  |
| Index child room sanitation | 6 | Swept & tidy | 277 | 204 | 73 (26.4) | 0.000 |
|  |  | Not swept, items scattered | 266 | 133 | 133 (50.0) |  |
|  |  | Other | 19 | 16 | 3 (15.8) |  |
| Index sleep on raised bed | 7 | No | 531 | 333 | 198 (37.3) | 0.663 |
|  |  | Yes | 30 | 20 | 10 (33.3) |  |
| Index child room organic matter | 8 | No | 257 | 181 | 76 (29.6) | 0.001 |
|  |  | Yes | 303 | 171 | 132 (43.6) |  |

^a^ p-values for chi2 of control vs. all cases; ^b^ household

## Table S4: Bivariable logistic regression for household risk factors for tungiasis, village as random effect

| **Variables** | **Categories** | **N^a^** | **OR^b^** | **95% CI^c^** | | **P^d^** |
| --- | --- | --- | --- | --- | --- | --- |
| Sex of index child | Female | 338 | 1 |  |  |  |
|  | Male | 230 | 1.83 | 1.12 | 2.99 | 0.016 |
| Sex of HH^e^ head | Male | 54 | 1 |  |  |  |
|  | Female | 515 | 1.21 | 0.50 | 2.92 | 0.671 |
| Age of HH head |  |  | 1.02 | 1.00 | 1.03 | 0.063 |
| Sex of caregiver | Male | 32 | 1 |  |  |  |
|  | Female | 537 | 0.59 | 0.19 | 1.84 | 0.363 |
| Age caregiver |  |  | 1.02 | 1.00 | 1.03 | 0.050 |
| Adult over 60 years in HH | No | 502 | 1 |  |  |  |
|  | Yes | 66 | 2.08 | 0.92 | 4.70 | 0.080 |
| Household head relationship to index child | Child | 432 | 1 |  |  |  |
|  | Grandchild | 63 | 1.68 | 0.72 | 3.89 | 0.227 |
|  | Other | 73 | 1.32 | 0.65 | 2.68 | 0.446 |
| Caregiver relationship to index child | Parent | 404 | 1 |  |  |  |
|  | Other | 165 | 1.50 | 0.87 | 2.58 | 0.147 |
| Socioeconomic status |  |  | 0.33 | 0.12 | 0.93 | 0.036 |
| Brew alcohol at home | No | 366 | 1 |  |  |  |
|  | Yes | 202 | 0.43 | 0.25 | 0.73 | 0.002 |
| Own land where house is | No | 185 | 1 |  |  |  |
|  | Yes | 384 | 0.99 | 0.59 | 1.66 | 0.975 |
| Water source | Piped or own well | 31 | 1 |  |  |  |
|  | Shared borehole | 415 | 0.69 | 0.04 | 12.42 | 0.801 |
|  | Unimproved/open | 119 | 0.2 | 0.01 | 4.28 | 0.306 |
| Collect water from short distance | No | 251 | 1 |  |  |  |
|  | Yes | 318 | 1.30 | 0.57 | 2.97 | 0.527 |
| Collect water from long distance | No | 307 | 1 |  |  |  |
|  | Yes | 262 | 0.69 | 0.31 | 1.53 | 0.366 |
| Number of jerricans water used daily | <1.5 | 154 | 1 |  |  |  |
|  | 1.5-3 | 324 | 0.62 | 0.33 | 1.17 | 0.138 |
|  | >3 | 87 | 0.43 | 0.20 | 0.95 | 0.037 |
| Bathing place | Built bathroom | 38 | 1 |  |  |  |
|  | Basin on compound | 378 | 1.53 | 0.89 | 3.96 | 0.383 |
|  | Makeshift bathroom | 147 | 0.99 | 0.36 | 2.77 | 0.992 |
| Caregiver foot wash frequency | Twice a day | 344 | 1 |  |  |  |
|  | Less often | 224 | 2.94 | 1.66 | 5.76 | <0.001 |
| Caregiver soap use | Always | 54 | 1 |  |  |  |
|  | Not always | 513 | 2.53 | 1.11 | 5.76 | 0.027 |
| Frequency wash young children | Twice a day | 329 | 1 |  |  |  |
|  | Once a day | 121 | 1.65 | 0.80 | 3.39 | 0.175 |
|  | Less often | 64 | 2.66 | 1.17 | 6.05 | 0.020 |
|  | Never | 52 | 1.74 | 0.80 | 3.82 | 0.165 |
| Toilet used | Latrine | 111 | 1 |  |  |  |
|  | Open defecation | 456 | 1.49 | 0.81 | 2.74 | 0.195 |
| Index child has another illness | Yes | 125 | 1 |  |  |  |
|  | No | 444 | 1.09 | 0.60 | 1.98 | 0.775 |
| Index child disability | No | 532 | 1 |  |  |  |
|  | Yes | 36 | 4.23 | 1.66 | 10.78 | 0.002 |
| Time spent talking with index child | None/a little | 366 | 1 |  |  |  |
|  | Some/ a lot | 203 | 1.47 | 0.80 | 2.69 | 0.214 |
| Hug and cuddle index child | Yes | 287 | 1 |  |  |  |
|  | No | 278 | 2.01 | 1.22 | 3.32 | 0.006 |
| Discipline style | Beat/ shout | 316 | 1 |  |  |  |
|  | Other | 252 | 0.74 | 0.45 | 1.24 | 0.256 |
| Know index child’s friends | No | 271 | 1 |  |  |  |
|  | Yes | 296 | 0.75 | 0.45 | 1.23 | 0.251 |
| Know parents of index child’s friends | No | 298 | 1 |  |  |  |
|  | Yes | 270 | 0.77 | 0.47 | 1.25 | 0.289 |
| Number of meals ate yesterday | 0 or 1 | 383 | 1 |  |  |  |
|  | 2 or 3 | 185 | 0.61 | 0.36 | 1.02 | 0.060 |
| Index child frequency wash feet | Twice a day | 388 | 1 |  |  |  |
|  | Less often | 159 |  |  |  |  |
|  | Don’t know | 21 | 1.62 | 0.44 | 6.03 | 0.468 |
| Index child soap use | Always | 28 | 1 |  |  |  |
|  | Not always | 521 | 6.41 | 1.65 | 24.9 | 0.007 |
|  | Don’t know | 19 | 5.91 | 0.87 | 40.09 | 0.069 |
| Caregiver education | None | 397 | 1 |  |  |  |
|  | Some | 172 | 0.72 | 0.43 | 1.20 | 0.208 |
| Caregiver marital status | Married | 447 | 1 |  |  |  |
|  | Other | 122 | 1.34 | 0.76 | 2.35 | 0.312 |
| Caregiver disability | No | 519 | 1 |  |  |  |
|  | Yes | 43 | 0.83 | 0.34 | 1.98 | 0.667 |
| Caregiver alcohol use | No | 78 | 1 |  |  |  |
|  | Yes | 484 | 1.39 | 0.64 | 3.01 | 0.411 |
| Caregiver occupation | Selling goods^g^ | 76 | 1 |  |  |  |
|  | None | 74 | 3.05 | 1.01 | 9.07 | 0.046 |
|  | Casual labor | 367 | 3.12 | 1.37 | 7.13 | 0.007 |
|  | Farming^h^ | 32 | 1.85 | 0.44 | 7.80 | 0.405 |
|  | Employment/other | 14 | 0.75 | 0.08 | 6.80 | 0.802 |
| HH is in a manyatta | No | 200 | 1 |  |  |  |
|  | Yes | 368 | 0.56 | 0.13 | 2.42 | 0.436 |
| HH in a shared homestead | No | 389 | 1 |  |  |  |
|  | Yes | 179 | 0.81 | 0.49 | 1.33 | 0.404 |
| Other HHs have jiggers | No | 106 | 1 |  |  |  |
|  | Yes | 72 | 15.61 | 6.28 | 38.75 | <0.001 |
| HH has a kitchen hut | No | 469 | 1 |  |  |  |
|  | Yes | 100 | 0.66 | 0.34 | 1.28 | 0.223 |
| HH has a separate sleeping hut | No | 506 | 1 |  |  |  |
|  | Yes | 63 | 1.44 | 0.69 | 3.02 | 0.332 |
| Number of adults in HH | 1-2 | 497 | 1 |  |  |  |
|  | >2 | 71 | 1.06 | 0.50 | 2.23 | 0.881 |
| Number of children under 5 years | 0 | 153 | 1 |  |  |  |
|  | 1-2 | 349 | 0.89 | 0.52 | 1.52 | 0.668 |
|  | >2 | 67 | 0.72 | 0.28 | 1.81 | 0.480 |
| Number of children 6 to 17 years | 0-2 | 359 | 1 |  |  |  |
|  | >2 | 210 | 1.15 | 0.70 | 1.89 | 0.582 |
| Floor adults sleep room | Hard clay | 405 | 1 |  |  |  |
|  | Loose clay/soil | 142 | 1.98 | 1.14 | 3.45 | 0.016 |
|  | Other | 8 | 1.52 | 0.27 | 8.44 | 0.632 |
| Number of children sleep in main house | 0 | 74 | 1 |  |  |  |
|  | 1-2 | 257 | 1.10 | 0.46 | 2.63 | 0.825 |
|  | >2 | 230 | 1.61 | 0.67 | 3.86 | 0.283 |
| Floor kids sleep on main house | Hard clay | 344 | 1 |  |  |  |
|  | Loose clay/soil | 125 | 2.39 | 1.32 | 4.33 | 0.004 |
|  | Other | 9 | 0.37 | 0.04 | 3.12 | 0.361 |
| Wall of main house | Sticks, leaves, grass | 411 | 1 |  |  |  |
|  | Mud/mixed mud & stone | 147 | 0.67 | 0.37 | 1.22 | 0.192 |
|  | Stone/ bricks /other | 2 | 11.72 | 0.12 | 1165.57 | 0.294 |
| Roof of main house | Iron sheets | 81 | 1 |  |  |  |
|  | Thatch of grass | 478 | 1.43 | 0.73 | 2.81 | 0.303 |
| State of repair main house | Good | 373 | 1 |  |  |  |
|  | Poor | 155 | 1.90 | 1.10 | 3.30 | 0.022 |
|  | Very poor (holes in walls & roof) | 33 | 2.56 | 0.84 | 7.79 | 0.097 |
| State of sanitation of main house | Swept & tidy | 287 | 1 |  |  |  |
|  | Not swept, items scattered | 257 | 2.36 | 1.43 | 3.88 | 0.001 |
|  | Other | 17 | 0.70 | 0.17 | 2.80 | 0.609 |
| Index child sleep room floor | Hard clay | 398 | 1 |  |  |  |
|  | Loose clay/soil | 155 | 1.86 | 1.07 | 3.21 | 0.027 |
|  | Other | 15 | 0.38 | 0.08 | 1.92 | 0.242 |
| Index sleep on raised bed | No | 531 | 1 |  |  |  |
|  | Yes | 30 | 0.66 | 0.23 | 1.90 | 0.444 |
| Index child room sanitation | Swept & tidy | 277 | 1 |  |  |  |
|  | Not swept, items scattered | 266 | 2.73 | 1.65 | 4.50 | <0.001 |
|  | Other | 19 | 0.56 | 0.12 | 2.67 | 0.470 |
| Index child room organic matter | No | 257 | 1 |  |  |  |
|  | Yes | 303 | 1.23 | 0.75 | 2.03 | 0.412 |

^a^ Number of households; ^b^ odds ratio; ^c^ confidence interval; ^d^ p-value; ^e^ head of household; ^g^ includes selling food, alcohol etc.; ^h^ crops or livestock.
